# Role and therapeutic potential of the NEDD4 family in breast cancer

**DOI:** 10.3389/fphar.2025.1587675

**Published:** 2025-06-04

**Authors:** Xiaoxiao Zhang, Sijie Li

**Affiliations:** Department of Breast Surgery, General Surgery Center, The First Hospital of Jilin University, Changchun, Jilin, China

**Keywords:** breast cancer, ubiquitin, Nedd4, Protac, TGF-β

## Abstract

The high incidence and mortality rates of breast cancer (BC) continue to pose a significant threat to patient survival and life expectancy. An increasing number of recent studies have demonstrated the crucial role of the ubiquitin-proteasome system (UPS) in cancer initiation and progression. Of particular significance is the aberrant expression of the NEDD4 family, which belongs to the HECT-type E3 ubiquitin ligases, in BC progression. This review synthesizes current knowledge about the interactions between the NEDD4 family and various tumor-related signaling pathways and their roles in BC biology, providing a theoretical foundation for exploring novel prognostic markers and treatment strategies.

## 1 Introduction

Breast cancer (BC) maintains the highest incidence and mortality rates among cancers in the global female population (Siegel et al., 2024). This disease poses a formidable threat to women’s health and survival. While chemotherapy remains the primary treatment option for patients with intermediate to advanced stages of BC, the development of chemotherapy resistance presents a persistent challenge for both healthcare providers and patients. This situation underscores the urgent need for novel therapeutic strategies.

**FIGURE 1:**
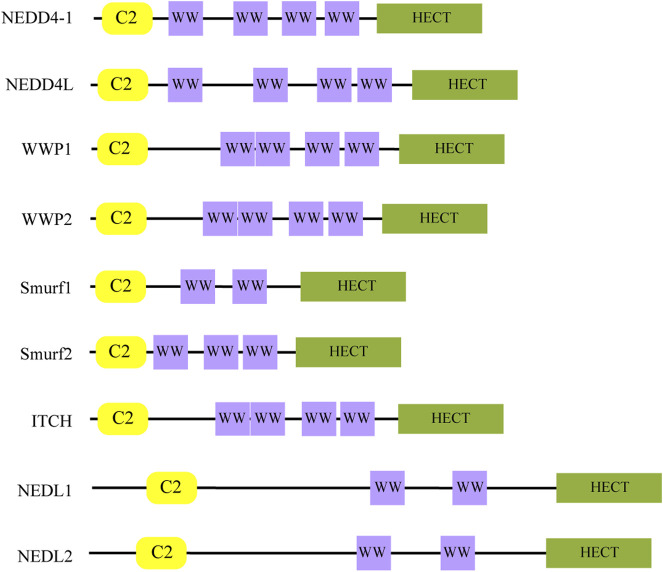
The structure of the NEDD4 family. The C2 domain is responsible for subcellular localization, the WW domain is responsible for substrate recognition, and the HECT domain is responsible for binding Ub.

Ubiquitination, a major form of post-translational protein modification, involves the covalent attachment of ubiquitin to target proteins through a cascade of enzymatic reactions (Swatek and Komander, 2016). The ubiquitin-proteasome system (UPS) comprises ubiquitin (Ub), E1 ubiquitin-activating enzyme, E2 ubiquitin-conjugating enzyme, E3 ubiquitin ligase, and the 26S proteasome. This system plays an essential role in cellular protein degradation and maintaining protein homeostasis (Schwartz and Ciechanover, 1999). Within this system, E3 ubiquitin ligases are crucial as they provide substrate specificity through binding to selective substrates (Hershko et al., 1983). The NEDD4 family, belonging to HECT E3 ubiquitin ligases, includes nine mammalian members: NEDD4-1, NEDD4L, WWP1/2, Smurf1/2, ITCH (AIP4), and NEDL1/2 (Ingham et al., 2004). As illustrated in Figure 1, these proteins share a characteristic three-component structure: an N-terminal C2 domain for cellular localization, two to four central WW domains that recognize substrate PY motifs, and a C-terminal HECT domain that binds Ub (Ingham et al., 2004; Chen and Matesic, 2007; Harvey and Kumar, 1999).

**FIGURE 2:**
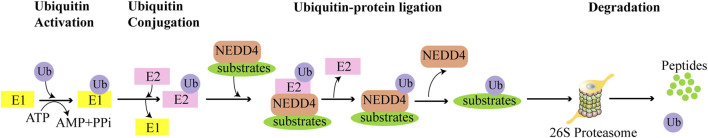
The NEDD4 family facilitates the degradation of substrates through UPS. Utilizing energy from ATP, the ubiquitin-activating enzyme E1 activates and binds to Ub. The activated Ub is then transferred from E1 to the ubiquitin-conjugating enzyme E2, which presents the Ub to the NEDD4 ligase. Subsequently, NEDD4 transfers the Ub to the specific substrate protein. Finally, the ubiquitin-labeled substrate is degraded by the 26S proteasome.

The NEDD4 family regulates substrate levels through the UPS and plays a role in a variety of cellular functions (Wang et al., 2020) (Figure 2). In addition, these proteins also influence cellular processes through mediating substrate ubiquitination and stabilization, facilitating targeted transport, and promoting lysosomal pathway-mediated degradation (Harvey and Kumar, 1999; Rotin and Kumar, 2009; Rotin and Prag, 2024). Studies have demonstrated elevated NEDD4-1 expression in BC tissues relative to normal breast tissues, with NEDD4-1 enhancing BC cell proliferation, migration, and stem cell characteristics (Jung et al., 2013; Wan et al., 2019; Jeon et al., 2020). Similarly, WWP1 shows upregulation in both BC cells and primary BC tissue, and its knockdown suppresses cell proliferation and activates apoptotic pathways (Chen et al., 2007; Nguyen Huu et al., 2008). In triple-negative breast cancer (TNBC), ITCH expression exceeds that found in luminal BC cells and normal mammary epithelial cells. Immunohistochemistry (IHC) analysis of human BC pathological tissue reveals significantly higher nuclear ITCH expression in TNBC compared to the luminal subtype. Furthermore, ITCH nuclear expression is elevated in metastatic lymph nodes compared to paired primary BC or normal breast tissue (Chang et al., 2019). This aberrant expression pattern of NEDD4 family members suggests their significant role in BC progression. To enhance our understanding of the role that the NEDD4 family plays in BC, this review provides an overview and summarizes the molecular mechanisms by which the NEDD4 family contributes to BC progression. Additionally, our analysis reveals that NEDD4 proteins may serve as potential prognostic markers for BC patients. Furthermore, we summarize the upstream regulatory mechanisms of the NEDD4 family, which provide insights for developing NEDD4-targeted strategies. This review addresses the knowledge gap regarding the multidimensional mechanisms of the NEDD4 family in BC.

## 2 Roles and molecular mechanisms of the NEDD4 family in BC

**FIGURE 3:**
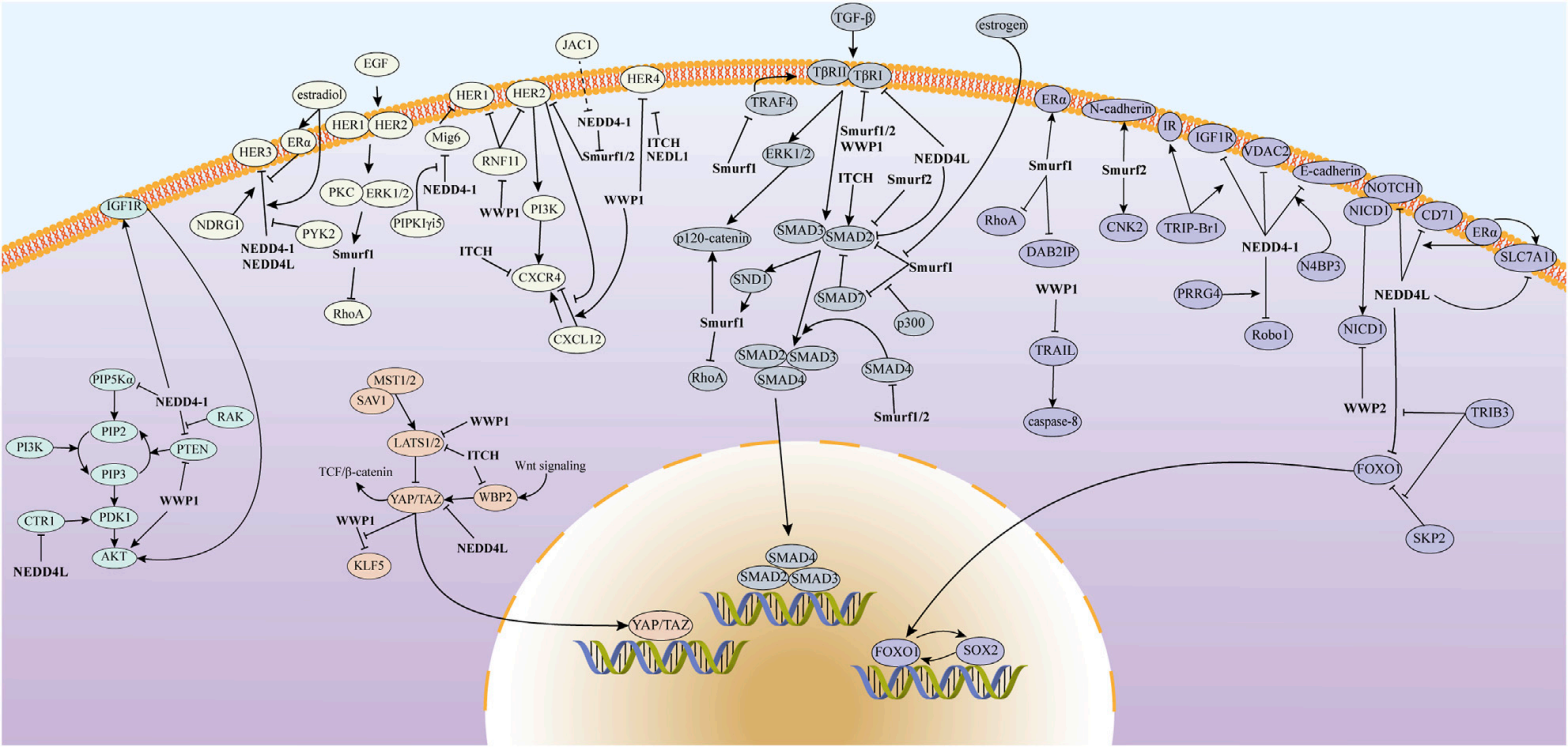
The mechanism of action of the NEDD4 family in BC involves the regulation of various signaling pathways, including PI3K/AKT, Hippo, EGFR, and TGF-β, which contribute to the malignant progression of the disease.

The substrate proteins of the NEDD4 family are involved in a range of classical signaling pathways associated with BC, including PI3K/AKT, Hippo, EGFR, TGF-β, NOTCH, and others (Figure 3). By interacting with these BC-associated pathways, NEDD4 proteins play important roles in BC tumorigenesis, cell proliferation, metastasis, ferroptosis, cancer stem cells (CSCs), and drug resistance.

### 2.1 Tumorigenesis

A combination of altered intrinsic genetic and epigenetic profiles, along with external signals, drives tumorigenesis (Zhang S. et al., 2024). The PI3K/AKT pathway, an intracellular signaling cascade, orchestrates cell metabolism, survival, proliferation, apoptosis, and motility through downstream substrate phosphorylation (Fresno Vara et al., 2004). Its dysregulation is strongly implicated in BC development (Guerrero-Zotano et al., 2016), and targeting PI3K/AKT pathway components has emerged as a significant therapeutic strategy (Nunnery and Mayer, 2020; Dey et al., 2017; Sharma et al., 2019). Phosphatase and tensin homolog (PTEN), a key tumor suppressor, negatively regulates the PI3K/AKT pathway (Stambolic et al., 1998). NEDD4-1 promotes carcinogenesis by mediating the proteasomal degradation of PTEN (Wang et al., 2007). By contrast, Rak, a nuclear tyrosine kinase, functions as a tumor suppressor in BC (Yim et al., 2009a). Yim et al. demonstrated that Rak attenuates the binding of PTEN to NEDD4-1 by phosphorylating PTEN, thereby inhibiting its degradation and preserving its anti-tumorigenic effects on BC cells (Yim et al., 2009b). Notably, NEDD4-1 does not affect PTEN ubiquitination or stability in mouse fibroblasts (Fouladkou et al., 2008), possibly due to the presence of proteins like Rak in non-cancerous cells, which impairs the regulatory effect of NEDD4-1 on PTEN (Yim et al., 2009b). Copper ions enter cells through the copper transporter protein CTR1 and bind to PDK1, activating the AKT signaling pathway and promoting carcinogenesis. NEDD4L counteracts this process by mediating CTR1 degradation via the UPS, thereby suppressing the PDK1-AKT pathway and inhibiting BC progression (Guo et al., 2021).

The Hippo signaling pathway, an evolutionarily conserved network, regulates crucial biological processes including tumorigenesis, cell proliferation, differentiation, survival, organ size, and tissue homeostasis through kinase cascades (Ma et al., 2019). In mammals, this pathway comprises key components including mammalian STE20-like kinases 1 and 2 (MST1/2), Salvador homolog 1 (SAV1), large tumor suppressor kinases 1 and 2 (LATS1/2), Yes-associated protein 1 (YAP), and WW domain-containing transcription regulator 1 (TAZ) (Ma et al., 2019). Upon pathway activation, MST1/2 and SAV1 form a complex that phosphorylates and activates LATS1/2, which then phosphorylates downstream transcriptional co-activators YAP and TAZ, repressing their nuclear translocation and transcriptional activity (Badouel and McNeill, 2011; Fu et al., 2022; Zhao et al., 2011; Zhao et al., 2007). The inactivation of LATS1/2 initiates basal-like BC that is dependent on the activity of YAP and TAZ (Kern et al., 2022). ITCH, the first identified negative regulator of LATS1, promotes cancer cell proliferation by mediating the degradation of LATS1 and enhancing the nuclear translocation of YAP (Ho et al., 2011). Its overexpression facilitates the transformation, survival, and epithelial-mesenchymal transition (EMT) of normal mammary epithelial cells by inhibiting LATS1 and stabilizing YAP (Salah et al., 2011). WW domain-binding protein 2 (WBP2) functions as a chaperone for WW domain proteins, exerting oncogenic effects by binding to YAP and TAZ through its PY motif (Chen et al., 2017; Chan et al., 2011). Interestingly, ITCH downregulates WBP2 via the UPS to inhibit TCF/β-catenin transcription, BC transformation, and tumorigenesis. By contrast, phosphorylation of tyrosine residues in WBP2 by the Wnt signaling pathway, along with the interaction between YAP/TAZ and WBP2, protects WBP2 from degradation by ITCH, thereby promoting the development of BC (Lim et al., 2016). These findings underscore how the role of NEDD4 proteins in BC varies significantly depending on their specific substrates.

### 2.2 Cell proliferation and tumor growth

Uncontrolled proliferation is a hallmark of cancer cells (Marcu, 2020). Type 1 insulin-like growth factor receptor (IGF1R), upregulated in BC, promotes tumor progression through activation of the PI3K/AKT signaling pathway (Zhou et al., 2022; Liu Z. et al., 2022). Wan et al. reported that NEDD4-1 promotes the proliferation of BC cells by activating the IGF1R/Akt pathway (Wan et al., 2019). While nuclear YAP promotes BC cell proliferation (Yang Y. et al., 2024), cytoplasmic YAP inhibits it through the activation of autophagy and is associated with a favorable prognosis for BC. NEDD4L promotes BC cell proliferation by mediating the degradation of cytoplasmic YAP (Guo et al., 2023). Similarly, WWP1 promotes BC cell proliferation by mediating LATS1 degradation through the UPS (Yeung et al., 2013).

The EGFR receptor tyrosine kinase family comprises four cell surface receptors: ErbB1/EGFR/HER1, ErbB2/HER2, ErbB3/HER3, and ErbB4/HER4 (Hsu and Hung, 2016). In BC, HER1, HER2, and HER3 exhibit a pro-carcinogenic effect (Hsu and Hung, 2016), whereas the role of HER4 is dual (Lucas et al., 2022), possibly due to the selective splicing of HER4 mRNA, which produces four variants: JM-a/CYT1, JM-a/CYT2, JM-b/CYT1, and JM-b/CYT2 (Veikkolainen et al., 2011). Mig6, a tumor suppressor, inhibits HER1 signaling through direct binding to HER1 (Zhang et al., 2007). NEDD4-1 promotes HER1 signaling by mediating polyubiquitination and proteasomal degradation of Mig6, although Type I γ-phosphatidylinositol phosphate 5-kinase i5 (PIPKIγi5) can counteract this by binding to NEDD4-1 and inhibiting Mig6 degradation (Sun et al., 2016). Among HER4 variants, CYT1 exhibits stronger anti-proliferative effects than CYT2 on breast epithelial cells (Wali et al., 2014). WWP1 specifically targets HER4 JM-a/CYT1 isoforms for ubiquitination and degradation, thereby modulating HER4 bioactivity in BC cells (Li et al., 2009; Feng et al., 2009). ITCH and NEDL1 also function as negative regulators of HER4 levels (Li et al., 2009).

NEDD4 proteins can also promote BC progression by inhibiting substrate ubiquitination. For instance, Smurf1 promotes ERα-positive BC cell proliferation by stabilizing ERα through inhibition of its polyubiquitination (Mayayo-Peralta et al., 2021; Yang et al., 2018). Similarly, Smurf2 enhances BC cell proliferation and invasion by preventing the polyubiquitination and proteasomal degradation of CNK2, a pro-cancer scaffolding protein (Serwe et al., 2023; David et al., 2014; David et al., 2018).

Conversely, the NEDD4 family can suppress BC proliferation by degrading oncogenic substrates. HER2, overexpressed in 15%–20% of BC cases and associated with malignant proliferation (Giaquinto et al., 2022), undergoes ubiquitination and proteasomal degradation by Smurf1/2 (Gu et al., 2022; Ren et al., 2021). NEDD4-1 inhibits the AKT pathway and BC proliferation by mediating UPS-dependent degradation of PIP5Kα, which normally activates the PI3K/AKT pathway through PIP2 generation (Choi et al., 2016; Tran et al., 2018). Krüppel-like factor 5 (KLF5) promotes BC growth and metastasis through multiple mechanisms, with its high expression correlating with poor prognosis (Wang H. et al., 2021; Zheng et al., 2009; Jia et al., 2016; Tong et al., 2006). WWP1 suppresses these effects by mediating KLF5 degradation through the UPS (Chen et al., 2005). However, TAZ/YAP can protect KLF5 from WWP1-mediated degradation by binding to KLF5 through its WW domain, thereby promoting BC cell proliferation, survival, and tumor growth (Zhao et al., 2012; Zhi et al., 2012). LATS1 counteracts this process by downregulating KLF5 through YAP inhibition (Zhi et al., 2012).

### 2.3 Migration and metastasis

Metastasis represents the leading cause of mortality in BC patients (Cancer Genome Atlas Network, 2012), with metastatic disease carrying a significantly worse prognosis (Allemani et al., 2018). EMT serves as a critical step in cancer cell metastasis (Foroni et al., 2012), with TGF-β acting as its primary inducer and a crucial mediator of BC metastasis (Xu et al., 2009; Imamura et al., 2012). The classical TGF-β pathway operates through transcription factors known as SMADs, which fall into three classes: receptor-regulated SMADs (R-SMADs: SMAD1, SMAD2, SMAD3, SMAD5, and SMAD8), the common-mediator SMAD (Co-SMAD: SMAD4), and inhibitory SMADs (I-SMADs: SMAD6 and SMAD7) (Derynck and Zhang, 2003; Padgett et al., 1998). R-SMADs serve as downstream signaling molecules of the TβR complex. TGF-β signaling initiates when TGF-β family ligand dimers form complexes with type II (TβRII) and type I (TβRI) receptors on the cell membrane, leading to TβRII phosphorylation and TβRI activation. Activated TβRI then recruits and activates R-SMADs, which form oligomers with SMAD4 and translocate to the nucleus for transcriptional regulation (Massagué, 2000; Deng et al., 2024). The pathway also promotes the expression of I-SMADs, which inhibit TGF-β signaling through multiple mechanisms: inhibiting R-SMAD phosphorylation, promoting dephosphorylation of TβRI, competing with activated R-SMADs to bind SMAD4, promoting oligomerization of R-SMADs, and recruiting E3 ubiquitin ligases to degrade pathway components (Miyazawa and Miyazono, 2017). NEDD4 proteins regulate TGF-β signaling by ubiquitinating SMADs and TGF-β receptors. Smurf1 and Smurf2, initially identified as regulatory molecules for SMADs and TGF-β receptors (Zhu et al., 1999; Zhang et al., 2001), along with WWP1 and NEDD4L, can be recruited by SMAD7 to activate TβRI. This interaction results in TβRI poly-ubiquitination and degradation (Ebisawa et al., 2001; Kavsak et al., 2000; Seo et al., 2004; Komuro et al., 2004; Kuratomi et al., 2005), a key event in the inhibition of TGF-β signaling. Estrogen suppresses BC metastasis by inhibiting the TGF-β signaling pathway through facilitating the formation of a ternary complex consisting of ERα, SMAD2/3, and Smurf1, leading to Smurf1-mediated SMAD2/3 degradation (Ito et al., 2010). Smurf2 mediates the ubiquitination and degradation of SMAD1 and SMAD2 with a preference for SMAD1 (Zhang et al., 2001), showing strong binding for phosphorylated TGF-β-activated SMAD2 compared to non-activated SMAD2 (Lin et al., 2000). Interestingly, the ubiquitination of SMAD2 by ITCH enhanced its binding to the TGF-β receptor, thereby positively regulating TGF-β signaling (Bai et al., 2004). NEDD4L also participates in SMAD2 regulation through its ubiquitination and subsequent degradation. In addition, during the formation of R-SMADS-SMAD4 oligomers, Smurf2 inhibits TGF-β signaling by mono-ubiquitinating SMAD4 (Zhou et al., 2017). Smurf1 mediates the polyubiquitination and degradation of SMAD4 with the involvement of SMAD7 (Morén et al., 2005). Although Smurf1 can also ubiquitinate and degrade SMAD7, the acetylation of SMAD7 at two lysine residues at its N-terminus, resulting from its interaction with the transcriptional co-activator p300, provides protection against this process (Grönroos et al., 2002).

TGF-β also activates non-classical pathways, including extracellular signal-regulated kinases (ERK), Rho-like GTPases, and PI3K/AKT pathways (Zhang, 2009). Within these pathways, p120-catenin, a central component of the cell adhesion junction (AJ) complex (Gumbiner, 2005), undergoes Smurf1-mediated monoubiquitination, while activation of ERK1/2 phosphorylates p120-catenin at the T900 locus, which further promotes the interaction of p120-catenin with Smurf1. The phosphorylation and monoubiquitination of p120-catenin are essential for AJ dissociation and BC metastasis, which is also a critical step in TGF-β-induced EMT (Wu et al., 2020). Tumor necrosis factor receptor-associated factor 4 (TRAF4) promotes BC metastasis by activating the TGF-β signaling pathway (Zhou et al., 2014). Li et al. demonstrated that Smurf1 mediates the polyubiquitination and the subsequent proteasomal degradation of TRAF4 (Li et al., 2010). In contrast, another study reported that the monoubiquitination of TRAF4 by Smurf1 facilitates its translocation to the cytoplasmic membrane and intercellular junctions, which is essential for Rac1 activation and BC cell migration (Wang et al., 2013). These findings suggest that differences in the type of NEDD4-mediated substrate ubiquitination can have varying effects on the substrate.

While dual targeting of HER2 and HER1 for degradation inhibits HER2-positive BC growth and metastasis and reduces drug resistance (Yang L. et al., 2024). Chen et al. reported that WWP1 promotes their expression indirectly by suppressing RNF11, their common negative regulator (Chen et al., 2008). Huang et al. found that NEDD4-1 mediates the degradation of HER3. Knockdown of NEDD4-1 enhances HER3-driven migration and proliferation of breast and prostate cancer cells, and promotes the growth of BC graft tumors. Notably, the downregulation of NEDD4-1, which leads to the accumulation of HER3, may increase the efficacy of anti-HER3 antibody therapy (Huang et al., 2015). In contrast, a study analyzing pathological tissue sections from BC patients revealed that high expression of NEDD4-1 is associated with elevated levels of cellular membrane-localized HER3 protein (Luhtala et al., 2018), suggesting that the degradation of HER3 by NEDD4-1 may be modulated by additional factors. For instance, estradiol has been shown to promote the degradation of HER3 by NEDD4-1 in BC cells, whereas ERα protects HER3 from degradation by NEDD4-1 (Suga et al., 2018). The regulation of HER3 protein levels by estradiol and ERα may contribute to resistance to endocrine therapy.

NEDD4-1 promotes the degradation of E-cadherin, a key event in EMT, and this process is further enhanced by N4BP3, which increases the E3 ligase activity of NEDD4-1 (Luo et al., 2022). In addition, NEDD4-1 mediates the degradation of Robo1, a tumor suppressor protein, facilitated by the transmembrane proline-rich γ-carboxyglutamic acid protein 4(PRRG4), which recruits NEDD4-1 to Robo1, thereby promoting BC metastasis (Zhang et al., 2020). DAB2IP is a known inhibitor of BC invasion and metastasis (Huang et al., 2023). Li et al. discovered that DAB2IP is degraded by Smurf1 in a ubiquitination-dependent manner, hence the depletion of Smurf1 results in the upregulation of DAB2IP, thereby inhibiting the proliferation and migration of breast and prostate cancer cells (Li et al., 2016). Smurf1 also regulates cell polarity and the formation of cellular protrusions by mediating the degradation of the small G protein RhoA via the UPS (Wang et al., 2003). Knockdown of Smurf1 leads to the accumulation of RhoA at the cell periphery, which inhibits BC cell migration (Sahai et al., 2007). Smurf2 has been implicated in promoting BC cell motility and invasiveness, potentially through the upregulation of N-cadherin at the protein level, independent of TGF-β signaling. The expression of E3 ligase-deficient mutants of Smurf2 inhibits BC metastasis (Jin et al., 2009).

The chemokine receptor CXCR4 is a G protein-coupled receptor that plays a key role in BC metastasis. Its activation is driven by the binding of its ligand CXCL12, which initiates downstream signaling that promotes metastatic progression (Müller et al., 2001; Yang et al., 2019). Following CXCL12 binding, CXCR4 undergoes internalization and is subsequently trafficked to lysosomes for degradation (Caballero et al., 2019). Both WWP1 and ITCH facilitate this process: WWP1 inhibits BC bone metastasis by enhancing CXCL12-mediated CXCR4 lysosomal translocation and degradation (Subik et al., 2012), while ITCH ubiquitinates CXCR4 at the plasma membrane for lysosomal degradation (Marchese et al., 2003). HER2 promotes BC metastasis in part by upregulating CXCR4 expression through activation of the PI3K signaling pathway and by inhibiting CXCL12-induced degradation of CXCR4. ITCH counteracts this effect by suppressing HER2-induced CXCR4 upregulation (Li et al., 2004). Kotb et al. reported an inverse correlation between ITCH and CXCR4 expression in tumor tissues from HER2-positive BC patients treated with trastuzumab. Moreover, elevated CXCR4 levels is associated with an increased risk of recurrence in BC patients undergoing trastuzumab therapy (Kotb et al., 2022).

The evolutionarily conserved NOTCH pathway, crucial for cell fate determination, operates through four mammalian receptors (NOTCH1, NOTCH2, NOTCH3, NOTCH4) (Schroeter et al., 1998). NOTCH1 often plays an oncogenic role in BC (Krishna et al., 2019), and its downregulation through reduced NOTCH1 intracellular domain (NICD1) levels suppresses BC development (Li et al., 2018; Shin et al., 2020). WWP2 contributes to this suppression by degrading NICD1 via the UPS, thereby inhibiting BC growth and metastasis (Zhang et al., 2023).

### 2.4 Ferroptosis

Ferroptosis is an iron-dependent form of programmed cell death characterized by excessive lipid peroxidation induced by reactive oxygen species, ultimately leading to tumor growth inhibition (Dixon et al., 2012). The process serves as one of the critical mechanisms by which ionizing radiation kills cancer cells (Lei et al., 2020). VDAC2, a voltage-dependent anion channel, positively regulates the sensitivity of cells to ferroptosis (Yang et al., 2020). NEDD4-1 controls ferroptosis by mediating the degradation of VDAC2 through the UPS. Natural bioflavonoids, such as RF-A, promote ferroptosis in BC cells by inhibiting the degradation of VDAC2 through binding to NEDD4-1 (Xie et al., 2021). Accumulating evidence suggests that the role of NEDD4L in ferroptosis in BC is time-dependent (Liu et al., 2021; Liu L. et al., 2022). SLC7A11, a key regulator of ferroptosis whose high expression is associated with poor prognosis in ER-positive BC, undergoes different regulation at distinct time points. After 12 h of ionizing radiation treatment, NEDD4L enhances ferroptosis by interacting with and degrading SLC7A11. However, during the early phase (4–12 h), ERα counteracts this effect by promoting the transcription of SLC7A11 (Liu et al., 2021). The transferrin receptor CD71 facilitates ferroptosis by promoting iron accumulation. At 48 h after ionizing radiation treatment, ERα inhibits ferroptosis by enhancing the binding of NEDD4L to CD71, leading to its degradation (Liu L. et al., 2022).

### 2.5 CSCs

CSCs possess self-renewal capacity and tumor-initiating potential, characteristics that significantly contribute to BC incidence, metastasis, and drug resistance (Wei and Lewis, 2015). The regulation of breast CSCs involves multiple NEDD4 family members through distinct pathways. Evidence for the role of NEDD4-1 in maintaining breast CSCs properties comes from studies showing reduced CSCs marker expression and activity in NEDD4-1-deficient BC cells. Specifically, NEDD4-1 knockdown significantly impairs mammosphere formation (Jeon et al., 2020). NOTCH1 receptor plays an important role in the formation and maintenance of breast CSCs (Simmons et al., 2012). NEDD4L suppresses NOTCH1-driven breast CSCs by mediating NOTCH1 degradation through the UPS (Guarnieri et al., 2018). FOXO1, an essential pluripotency factor for cellular stemness (Zhang et al., 2011), undergoes regulation by both NEDD4L and SKP2 through ubiquitination and its subsequent degradation. However, TRIB3 counteracts this regulation by protecting FOXO1 from NEDD4L and SKP2-mediated degradation. The resulting elevated FOXO1 levels increase SOX2 transcription, which in turn enhances FOXO1 transcription, establishing a positive feedback loop that promotes breast CSC maintenance (Yu et al., 2019).

### 2.6 Resistance to drugs

Drug resistance in BC treatment remains a significant clinical challenge. WWP1 contributes to this resistance by inhibiting PTEN function through multiple mechanisms: mediating the polyubiquitination of PTEN, preventing its dimerization, and blocking its membrane recruitment (Lee et al., 2019). Kishikawa et al. reported that WWP1’s regulation of PTEN reduces the effectiveness of PI3K inhibitors in BC treatment, while WWP1 inhibition restores PTEN function and suppresses the PI3K/AKT pathway (Kishikawa et al., 2021). WWP1 may also influence PI3K/AKT signaling through PTEN-independent mechanisms, as evidenced by Wang et al.'s finding that WWP1 overexpression activates the PI3K/AKT pathway and reduces BC sensitivity to paclitaxel without significantly altering PTEN protein levels (Wang L. et al., 2021).

An elevated insulin receptor (IR)/IGF1R ratio is associated with poor prognosis in BC (Gallagher et al., 2020). Ulanet et al. discovered that this high ratio renders BC cells insensitive to IGF1R antibody (A12) treatment, while knockdown of IR significantly enhances the inhibitory effect of A12 on BC cells (Ulanet et al., 2010). The oncogenic protein TRIP-Br1 influences this ratio by inhibiting ubiquitination and degradation of IR while cooperating with NEDD4-1 to promote IGF1R degradation via the UPS. This interaction increases the IR/IGF1R ratio at the protein level, promoting both BC cell proliferation and drug resistance (Nguyen et al., 2022). These findings appear to contrast with experimental results reported by Wan et al., which indicated that NEDD4-1 upregulates IGF1R (Wan et al., 2019), suggesting that NEDD4-1 may regulate IGF1R and its downstream signaling through alternative mechanisms, contributing to BC progression. For example, a previous study demonstrated that NEDD4-1 positively regulates IGF1R by modulating the function of the articulin Grb10 in mouse embryonic fibroblasts (Cao et al., 2008).

TNBC resistance to EGFR inhibitors frequently involves HER3 upregulation (Tao et al., 2014). Verma et al. revealed that the non-receptor tyrosine kinase PYK2 promotes this resistance by preventing NEDD4-1/NEDD4L from interacting with and degrading HER3. N-myc downstream-regulated gene 1 (NDRG1) enhances HER3 degradation by promoting its interaction with NEDD4-1/NEDD4L. Importantly, targeting PYK2 expression or activity can reduce TNBC resistance to EGFR inhibitors (Verma et al., 2017).

Tumor necrosis factor (TNF)-related apoptosis-inducing ligand (TRAIL), which induces tumor-specific apoptosis through caspase-8 activation (de Miguel et al., 2016), faces resistance mechanisms involving WWP1. Through its E3 ligase activity, WWP1 inhibits the caspase-8-dependent exogenous apoptosis pathway, conferring TRAIL resistance. While WWP1 knockdown enhances TRAIL effectiveness, the mechanism remains unclear as caspase-8 is not a direct WWP1 substrate (Zhou et al., 2012), suggesting indirect regulation of TRAIL-caspase-8 signaling.

In summary, NEDD4 proteins are intricately linked to BC oncogenesis and progression through various mechanisms. It is important to acknowledge that the role of the NEDD4 family in BC predominantly depends on their regulation of the biological functions of their substrates. Considering the diversity of these substrates and the concurrent activity of multiple signaling pathways, the effects of the NEDD4 family on BC cell phenotypes are often dualistic. We summarize the roles and mechanisms of the NEDD4 family in BC in Table 1.

**TABLE 1:** Roles of the NEDD4 family in BC.

Proteins	Substrates	Mechanisms	Effects	Experimental model	References
NEDD4-1	PTEN	Mediating the polyubiquitination and proteasomal degradation of PTEN	↑Tumorigenesis	Cell lines; Cell derived xenograft (CDX) models	Yim et al. (2009b)
NEDD4L	CTR1	Mediating the degradation of CTR1 via the UPS to suppresses the PDK1-AKT pathway	↓Tumorigenesis	Cell lines; Breast tissues; CDX models	Guo et al. (2021)
ITCH	LATS1	Inhibiting LATS1 and stabilizing YAP	↑Tumorigenesis	Cell lines	Ho et al. (2011), Salah et al. (2011)
ITCH	WBP2	Mediating the degradation of WBP2 via the UPS, which inhibits TCF/β-catenin transcription	↓Tumorigenesis	Cell lines; CDX models	Lim et al. (2016)
NEDD4-1	N/A	Upregulating positively IGF1R/Akt pathway	↑Cell proliferation	Cell lines; Breast tissues	Wan et al. (2019)
NEDD4L	YAP	Mediating the ubiquitination and degradation of YAP, hence downregulating cytoplasmic YAP	↑Cell proliferation	Cell lines; Breast tissues	Guo et al. (2023)
WWP1	LATS1	Mediating the degradation of LATS1 via the UPS	↑Cell proliferation	Cell lines	Yeung et al. (2013)
Smurf1	N/A	Stabilizing ERα by inhibiting K48-dependent polyubiquitination on ERα proteins	↑Cell proliferation	cell lines; CDX models	Yang et al. (2018)
Smurf2	N/A	Upregulating CNK2 by inhibiting the polyubiquitination and proteasomal degradation of CNK2	↑Cell proliferation	Cell lines; Breast tissues	David et al. (2014), David et al. (2018)
NEDD4-1	PIP5Kα	Mediating the degradation of PIP5Kα via UPS, which inhibits the AKT pathway	↓Cell proliferation	Cell lines	Tran et al. (2018)
WWP1	KLF5	Mediating the degradation of KLF5 via the UPS	↓Cell proliferation and survival ↓Tumor growth	Cell lines; CDX models	Chen et al. (2005), Zhao et al. (2012), Zhi et al. (2012)
Smurf1	SMAD2/3	Mediating the degradation of SMAD2/3 to inhibit the TGF-β signaling pathway	↓Metastasis	Cell lines	Ito et al. (2010)
Smurf1	p120-catenin	Mediates the monoubiquitination of p120-catenin and dissociation of AJ	↑ EMT and metastasis	Cell lines; Breast tissues; CDX models	Wu et al. (2020)
Smurf1	TRAF4	Mediating the monoubiquitination of TRAF4, which promotes the translocation of TRAF4 to the cytoplasmic membrane and intercellular junctions	↑Cell migration	Cell lines	Wang et al. (2013)
NEDD4-1	HER3	Mediating the degradation of HER3	↓Cell migration and proliferation ↓Tumor growth	Cell lines; CDX models	Huang et al. (2015)
NEDD4-1	E-cadherin	Mediating the ubiquitination and degradation of E-cadherin	↑Metastasis	Cell lines	Luo et al. (2022)
NEDD4-1	Robo1	Mediating the ubiquitination and degradation of Robo1	↑Metastasis	Cell lines	Zhang et al. (2020)
Smurf1	DAB2IP	Mediating the degradation of DAB2IP in a ubiquitination-dependent manner	↑Cell proliferation and migration	Cell lines	Li et al. (2016)
Smurf1	RhoA	Mediating the degradation of RhoA via the UPS	↑Cell migration	Cell lines; CDX models	Wang et al. (2003), Sahai et al. (2007)
Smurf2	N/A	Mediating the upregulation of N-cadherin	↑Metastasis	Cell lines; breast tissues; CDX models	Jin et al. (2009)
WWP1	N/A	Enhancing lysosomal translocation and degradation of CXCR4 by CXCL12	↓BC bone metastasis	Cell lines; Breast tissues; CDX models	Subik et al. (2012)
ITCH	CXCR4	Mediating the ubiquitination and degradation of CXCR4	↓Metastasis	Cell lines	Marchese et al. (2003), Li et al. (2004)
WWP2	NICD1	Mediating the degradation of NICD1 via the UPS	↓BC growth and metastasis	Cell lines; CDX models	Zhang et al. (2023)
NEDD4-1	VDAC2	Mediating the degradation of VDAC2 via the UPS	↓Ferroptosis	Cell lines	Yang et al. (2020), Xie et al. (2021)
NEDD4L	SLC7A11	Mediating the degradation of SLC7A11 via the UPS	↑Ferroptosis	Cell lines	Liu et al. (2021)
NEDD4L	CD71	Mediating the degradation of CD71 via the UPS	↓Ferroptosis	Cell lines	Liu et al. (2022b)
NEDD4-1	N/A	Maintaining the mammary CSCs characteristics	↑CSCs	Cell lines	Jeon et al. (2020)
NEDD4L	NOTCH1	Mediating the degradation of NOTCH1 via the UPS	↓CSCs	Cell lines	Simmons et al. (2012), Guarnieri et al. (2018)
NEDD4L	FOXO1	Mediating the degradation of FOXO1	↓CSCs	Cell lines	Yu et al. (2019)
WWP1	PTEN	Inhibiting PTEN dimerization, membrane recruitment, and anticarcinogenic effect by mediating polyubiquitination of PTEN	↑Resistance to PI3K inhibitors	Cell lines; Patient derived xenograft (PDDX) models	Lee et al. (2019), Kishikawa et al. (2021)
WWP1	N/A	Upregulating the phosphorylation levels of AKT, which activates the PI3K/AKT pathway	↑Resistance to paclitaxel	Cell lines; Breast tissues; PDX models	Wang et al. (2021b)
NEDD4-1	IGF1R	Mediating the degradation of IGF1R via the UPS	↑Resistance to anticancer drugs	Cell lines	Nguyen et al. (2022)
NEDD4-1/NEDD4L	HER3	Mediating the degradation of HER3	↓Resistance to EGFR inhibitors	Cell lines	Verma et al. (2017)
WWP1	N/A	Suppressing apoptosis by inhibiting the caspase-8-dependent exogenous apoptosis pathway	↑Resistance to TRAIL	Cell lines	Zhou et al. (2012)

## 3 NEDD4 family and prognosis in BC patients

Natori et al. reported that low levels of NEDD4-1 mRNA are associated with longer disease-free survival (DFS) and overall survival (OS) in a cohort of HR-positive BC patients treated with endocrine therapy. This finding may be attributed to the fact that NEDD4-1 negatively regulates ERα expression; consequently, low levels of NEDD4-1 lead to an upregulation of ERα, which enhances the sensitivity of cancer cells to endocrine therapy (Natori et al., 2023). The Kaplan-Meier (KM) plotter indicated that patients with high NEDD4-1 expression exhibit lower survival rates compared to those with low NEDD4-1 expression in highly aggressive BC populations, specifically HER2-positive and TNBC. Furthermore, NEDD4-1 expression is elevated in TNBC cells relative to ER-positive or HER2-positive BC cells (Jeon et al., 2020). Wan et al. demonstrated that NEDD4-1 is highly expressed in BC and is correlated with tumor size, ER status, PR status, and lymph node status. Furthermore, the positive rate of NEDD4-1 expression increases with tumor progression. The 10-year OS and DFS of patients with positive NEDD4-1 expression are significantly lower than those of BC patients with negative NEDD4-1 expression. Subgroup analysis revealed that NEDD4-1 had the most significant prognostic impact on ER-negative BC (Wan et al., 2019), suggesting its potential as a marker for poor prognosis.

Compared to normal tissue, the expression of NEDD4L is downregulated in BC tissue (Guo et al., 2022). Bioinformatics analysis of the TCGA dataset revealed that the expression of NEDD4L in BC tissues and metastatic cancer tissues is significantly lower than in normal tissues. KM analysis of distant relapse-free survival (DRFS) based on the GEO dataset (GSE22219) confirmed that low expression of NEDD4L is associated with a poor prognosis (Guarnieri et al., 2018). Additionally, the KM Plotter indicated that low NEDD4L expression is associated with shorter OS and recurrence-free survival (RFS) in BC (Guo et al., 2022). Given that most NEDD4L substrates promote oncogenesis, NEDD4L shows promise as a valuable prognostic indicator.

WWP1 expression is elevated in TNBC tissue compared to adjacent normal tissue, with high WWP1 levels correlating with shorter OS in TNBC patients (Wang L. et al., 2021). Nguyen Huu et al. classified the samples into 4 categories based on the distribution and intensity of IHC staining of WWP1 in BC tissues: Category 1 exhibited no or low staining; Category 2 displayed heterogeneous staining resembling the staining pattern of normal breast tissue, characterized by moderate or strong nuclear staining and minimal cytoplasmic staining in approximately 50% of tumor cells; Category 3 showed homogeneous, moderate to intense nuclear staining; and Category 4 presented homogeneous, moderate to intense nuclear staining along with cytoplasmic staining. The analysis indicated that the WWP1 staining pattern correlates with BC prognosis, with Category 1 staining associated with the worst prognosis and Category 3 staining linked to the best prognosis. This suggests that the role of WWP1 in BC may be influenced by its subcellular localization (Nguyen Huu et al., 2008). Chen et al. reported that cytoplasmic WWP1 expression is positively correlated with the expression of ERα and IGF1R proteins in primary BC tissues (Chen et al., 2009).

WWP2 expression is downregulated in BC tissues compared to matched adjacent tissues. High WWP2 expression is negatively correlated with the levels of NICD1 and Ki-67, a marker of cellular proliferation, while exhibiting a positive correlation with the expression of E-cadherin. Furthermore, low WWP2 expression is associated with reduced OS in BC patients (Zhang et al., 2023).

In summary, NEDD4 proteins are frequently abnormally expressed in BC and are associated with its prognosis (see Table 2). This suggests that NEDD4 proteins may serve as novel prognostic markers for BC.

**TABLE 2:** Prognostic roles of the NEDD4 family in BC.

Proteins	Detection levels	Data source	BC patient sample size(n)	Pathological subtypes	Relevant pathologic parameters	Prognostic indicators	References
NEDD4-1	mRNA	clinical sample	143	HR+ and HER2-		DFS (*p* = 0.048); OS (*p* = 0.022)	Natori et al. (2023)
NEDD4-1	mRNA	Kaplan-Meier plotter	618	TNBC		Survival rate (*p* = 0.015)	Jeon et al. (2020)
			251	HER2+ and ER-		Survival rate (*p* = 0.0084)	
NEDD4-1	protein	clinical sample	455		tumor size (*p* = 0.030); nodal status (*p* = 0.001); ER status (*p* = 0.035); PR status (*p* = 0.023); TNM stages (stage II vs. І, *p* = 0.003; stage III vs. І, *p* < 0.001; stage III vs. II, *p* = 0.023)		Wan et al. (2019)
			297			DFS (*p* = 0.0011); OS (*p* = 0.0024)	
			99	ER-		OS (*p* = 0.0204)	
			219	HER2-		OS (*p* = 0.0355)	
	mRNA	TCGA	123	ER-		DMFS (*p* = 0.00333); OS (*p* = 0.04835)	
NEDD4L	mRNA	GEO	216			DRFS (*p* = 0.0019)	Guarnieri et al. (2018)
NEDD4L	mRNA	Kaplan-Meier plotter	1879			OS (*p* = 0.00027)	Guo et al. (2022)
			4929			RFS (*p* < 0.00001)	
WWP1	protein	clinical sample	90	TNBC		OS (*p* = 0.015)	Wang et al. (2021b)
WWP1	protein	clinical sample	419			DFS (*p* < 0.05)	Nguyen Huu et al. (2008)
			312			OS (*p* = 0.04)	
WWP2	mRNA protein	clinical sample	67			OS (*p* = 0.0043)	Zhang et al. (2023)

*p* < 0.05, statistically significant.

## 4 Regulation of NEDD4 expression in BC

**FIGURE 4:**
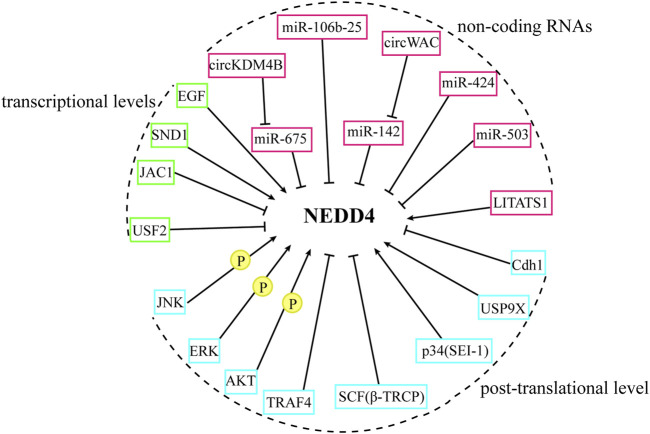
The regulation of NEDD4 proteins. The NEDD4 family are regulated from the transcriptional level, non-coding RNA, and post-transcriptional level.

The NEDD4 family, while functioning as E3 ubiquitin ligases to regulate substrate levels, is itself subject to multiple regulatory mechanisms. Understanding these regulatory pathways, summarized in Figure 4, may facilitate the development of strategies that target the NEDD4 family.

### 4.1 Regulation of the NEDD4 family at the transcriptional levels

Upstream stimulatory factor 2(USF2) suppresses Smurf1/2 transcriptional activity by binding to their promoter regions, consequently promoting TGF-β signal transduction (Tan et al., 2019). Within this regulatory network, NEDD4-1 acts as a negative regulator upstream of Smurf1 (Ren et al., 2021). The cancer suppressor protein JWA, known to negatively regulate HER2 expression in gastric cancer, influences this pathway through the JWA gene activating compound 1 (JAC1). JAC1 downregulates NEDD4-1 mRNA expression, thereby activating Smurf1-mediated proteasomal degradation of HER2 and inhibiting the proliferation of HER2-positive BC cells and tumor growth (Ren et al., 2021). TGF-β signaling activates Smad2/3 to upregulate the oncoprotein SND1, which enhances both mRNA and protein expression of Smurf1. This increased Smurf1 expression leads to greater RhoA degradation, promoting BC metastasis (Yu et al., 2015). EGF promotes BC migration through HER1 and HER2 heterodimerization and subsequent pathway activation (Dittmar et al., 2002). Kwon et al. reported that EGF increases Smurf1 expression at both mRNA and protein levels through HER1 and HER2-induced activation of Protein Kinase C and ERK1/2 signaling, resulting in RhoA downregulation and enhanced BC cell migration and invasion (Kwon et al., 2013).

### 4.2 Regulation of the NEDD4 family by non-coding RNAs

Non-coding RNAs are being explored for their role in BC (Crudele et al., 2020). Among them, microRNAs (miRNAs), a class of endogenous small non-coding RNAs, inhibit target gene translation by binding to the 3′UTR of target mRNAs (Zamore and Haley, 2005). Long non-coding RNAs (lncRNAs) and circular RNAs (circRNAs) can function as miRNA sponges, thereby modulating target gene expression (Beňačka et al., 2024).

NEDD4L-mediated degradation of PI3K through the UPS inhibits PI3K/AKT signaling (Wang et al., 2016). As a target gene of miR-675, NEDD4L is regulated by circular RNA circKDM4B, which acts as an effective sponge for miR-675. This regulation leads to the upregulation of NEDD4L and enhanced PI3K degradation, ultimately inhibiting angiogenesis and metastasis in BC (Guo et al., 2022). Similarly, the miR-106b-25 cluster targets NEDD4L, promoting breast CSCs by activating NOTCH1 through NEDD4L downregulation (Guarnieri et al., 2018). WWP1, a target gene of miR-142, influences PI3K/AKT signaling. Circular RNA circWAC functions as a sponge for miR-142, protecting WWP1 expression and thereby promoting PI3K/AKT pathway activation in TNBC (Wang L. et al., 2021). The regulation of TGF-β signaling involves miR-424 and miR-503, which target both Smurf2 and SMAD7. These miRNAs enhance TGF-β signaling and BC metastasis by reducing Smurf2 and SMAD7 expression (Li et al., 2014). Additionally, the cytoplasmic lncRNA LITATS1, upregulated by TGF-β signaling, binds to the WW1 structural domain of Smurf2, promoting its cytoplasmic retention. This interaction enhances the polyubiquitination and degradation of TβRIs, resulting in the inhibition of TGF-β signaling and the migration of BC cells (Fan et al., 2023). Thus, a complex interplay exists between non-coding RNAs, the NEDD4 family, and tumor-associated signaling pathways. However, related studies remain limited, and further exploration is essential to achieving a deeper understanding of the roles and molecular mechanisms of ncRNAs and the NEDD4 family in BC.

### 4.3 Regulation of the NEDD4 family at the post-translational level

#### 4.3.1 Self-ubiquitination and ubiquitination

The interaction between the C2 or WW structural domains and the HECT structural domain maintains NEDD4 in an autoinhibited state, reducing its E3 ubiquitin ligase activity (Wan et al., 2011; Wiesner et al., 2007; Wang et al., 2019; Zhu et al., 2017). The tumor suppressor Cdh1 enhances this autoinhibition in WWP2 by promoting interaction between its C2 and HECT domains. This mechanism protects PTEN from WWP2-mediated degradation, thereby inhibiting AKT signaling and BC growth (Liu et al., 2016). Xie et al. identified USP9X as a Smurf1-interacting deubiquitinase that binds to Smurf1 via its carboxyl terminus, preventing its self-ubiquitination and subsequent proteasomal degradation. USP9X depletion reduces BC migration by downregulating Smurf1 (Xie et al., 2013). NEDD4-1 can promote PTEN nuclear translocation through monoubiquitination, where nuclear PTEN shows increased stability and enhances apoptosis by inhibiting AKT (Trotman et al., 2007). The oncoprotein p34 (SEI-1), encoded by the *SEI-1* gene, stabilizes NEDD4-1 by preventing its self-ubiquitination and degradation, thereby enhancing PTEN polyubiquitination and degradation. Conversely, p34 (SEI-1) knockdown increases PTEN monoubiquitination and nuclear translocation (Hong et al., 2014). Jung et al. also reported that p34 (SEI-1) promotes AKT phosphorylation and reduces PTEN levels by upregulating NEDD4-1 expression in BC, although they observed increased PTEN nuclear translocation following the overexpression of p34 (SEI-1) (Jung et al., 2013). Several possible explanations exist for the discrepancy between the results of the two studies mentioned above: 1. The regulation of PTEN by p34 (SEI-1) may not solely depend on NEDD4-1 but could involve other factors; 2. A negative feedback mechanism may operate in cells at very low levels of PTEN to facilitate its translocation into the nucleus; and 3. Translocated nuclear PTEN may be degraded through alternative pathways. For example, it has been demonstrated that the E3 ubiquitin ligase FBXO22 induces the ubiquitination and degradation of nuclear PTEN at the lysine 221 site, without affecting cytoplasmic PTEN (Ge et al., 2020). Additionally, NEDD4-1 itself is a substrate of the E3 ubiquitin ligase complex SCF(β-TRCP). Casein kinase Iδ phosphorylates NEDD4-1 at S347/S348, promoting its binding to SCF(β-TRCP) and subsequent proteasomal degradation. Mutant NEDD4-1 that evades this degradation enhances the growth and migration of breast and prostate cancer cells by reducing PTEN levels (Liu et al., 2014). Smurf2 can be recruited to activate TβRI through its interaction with SMAD7, leading to the polyubiquitination and degradation of TβRI, a crucial event in the inhibition of TGF-β signaling (Kavsak et al., 2000). TRAF4, functioning as an E3 ubiquitin ligase, targets Smurf2 for degradation (Li et al., 2019; Zhang et al., 2013), thereby stabilizing TβRI on BC cell membranes and enhancing TGF-β signaling (Zhang et al., 2013). The mitosis-associated protein Eg5 maintains cell proliferation (El-Nassan, 2013). Smurf2 downregulates Eg5 levels through polyubiquitination (Hao et al., 2022). TRAF4 inhibits apoptosis in BC cells and promotes cell proliferation by both preventing Smurf2-Eg5 binding and targeting Smurf2 for ubiquitination, resulting in elevated Eg5 levels (Hao et al., 2022). Additionally, Smurf2 regulates Smurf1 through ubiquitination and degradation, thereby inhibiting BC cell migration, while Smurf1 cannot degrade Smurf2 (Fukunaga et al., 2008).

#### 4.3.2 Phosphorylation

Phosphorylation of NEDD4 proteins enhances their inhibitory effects on substrates. AKT-mediated phosphorylation of Smurf1 increases its stability and amplifies DAB2IP downregulation (Li et al., 2016). AKT also phosphorylates ITCH at Ser257, facilitating its nuclear translocation, where it inhibits 53BP1 foci formation by ubiquitinating histone H1.2 at K46. This mechanism renders BC cells resistant to replicative stress and DNA damage, promoting tumor growth and metastasis (Chang et al., 2019). During TGF-β-induced EMT, ERK phosphorylates Smurf1 at threonine 223, enhancing its ability to polyubiquitinate and degrade RhoA, thereby promoting EMT and BC metastasis (Zheng et al., 2022). C-FLIP, which negatively regulates the TRAIL-caspase-8 apoptotic pathway (Zhang et al., 2004; Haag et al., 2011), undergoes proteasomal degradation when JNK phosphorylates and activates ITCH during TNFα signaling, leading to apoptosis (Chang et al., 2006). Notably, endocrine-resistant ER-positive BC cells exhibit sensitivity to TRAIL-induced cell death due to increased degradation of c-FLIP, resulting from JNK-mediated enhancement of ITCH phosphorylation (Piggott et al., 2018).

## 5 Targeting NEDD4 for BC treatment

The aberrant expression of NEDD4 family members and their association with BC cell malignancy suggest their potential as therapeutic targets. NEDD4 proteins primarily influence tumor progression through their E3 ubiquitin ligase activity, mediating substrate ubiquitination and proteasomal degradation to exert both tumor-promoting and tumor-suppressive effects. Therefore, modulating UPS may be a promising strategy for BC therapy.

Proteasome inhibitors have demonstrated promise in BC therapy by preventing E3 ubiquitin ligase-mediated substrate degradation *in vivo* (Shen et al., 2015; Agyin et al., 2009; Schmid et al., 2008; Schwartz et al., 2021). However, their lack of specificity remains a limitation. Proteolysis-targeting chimeric (PROTAC) technology offers a more targeted approach. PROTACs employ specially designed linker molecules to connect the ligand of the E3 ubiquitin ligase to the ligand of the protein of interest (POI). Upon cellular entry, PROTACs facilitate the interaction between an E3 ubiquitin ligase and the POI, triggering ectopic ubiquitylation and subsequent proteasomal degradation of the POI (Neklesa et al., 2017). This enables specific targeting of oncogenic substrates of NEDD4 proteins. For example, a novel PI3K-PROTAC that induces the degradation of PI3K-p110α through CRBN and the proteasome has been found to specifically inhibit BC cell lines harboring the PIK3CA mutation, while enhancing HER2-positive BC sensitivity to lapatinib (Zhang H. et al., 2024). Similarly, Gough et al. demonstrated that the oral PROTAC drug Vepdegestrant, which connects CRBN and ER, promotes ER degradation and inhibits tumor growth in a preclinical BC model (Gough et al., 2024). Phase I/II clinical studies have shown that Vepdegestrant monotherapy demonstrates good tolerability and clinical efficacy in previously treated ER+/HER2- advanced BC patients (Iwata et al., 2025; Hamilton et al., 2024). A large Phase III clinical study investigating the safety and efficacy of Vepdegestrant in advanced BC (NCT05654623) is currently ongoing. However, PROTACs design currently utilizes only a limited number of E3 ligases, necessitating additional preclinical and clinical studies to evaluate their therapeutic potential in BC. Molecular glue compounds function similarly to PROTACs but offer potential advantages through their lower molecular weight, which may improve oral bioavailability and cellular permeability (Zhao et al., 2022). For example, the small molecule C1, designed by Zhong et al. as a molecular glue, enhances eEF2K-βTRCP interaction, facilitating eEF2K degradation through the ubiquitin-proteasome pathway and downregulates eEF2K protein expression, thereby inhibiting TNBC tumors (Zhong et al., 2024). In conclusion, both classes of drugs—PROTAC and molecular glue—provide innovative strategies for targeting the substrates of E3 ubiquitin ligase in the treatment of BC.

The development of small-molecule inhibitors and agonists targeting the NEDD4 family may provide additional therapeutic options for BC patients. For instance, Indole-3-Carbinol, a natural inhibitor of WWP1, enhances the anti-cancer effects of PTEN by inhibiting WWP1 (Lee et al., 2019). High-throughput screening identified the antidepressant clomipramine as an ITCH inhibitor that limits cancer cell growth and enhances chemotherapy efficacy by blocking cancer cell autophagy (Rossi et al., 2014). The various upstream NEDD4 regulators discussed earlier may also represent potential therapeutic targets, though their other roles in BC must be carefully considered.

TNBC, the most aggressive subtype of BC, presents particular therapeutic challenges due to its resistance to endocrine therapy and HER2-targeted treatments, often leaving chemotherapy as the primary option (Cancer Genome Atlas Network, 2012). Drug resistance and tumor heterogeneity further limit the effectiveness of chemotherapy in some TNBC patients (Mayer et al., 2014). Therefore, it is crucial to identify more effective treatments for TNBC. Aberrant activation of multiple signaling pathways, including PI3K/AKT/mTOR, EGFR, TGF-β, and NOTCH, contributes to TNBC progression (Mayer et al., 2014; Jamdade et al., 2015). While ongoing clinical studies of PI3K inhibitors, AKT inhibitors, mTOR inhibitors, and EGFR inhibitors, in combination with other drugs, have shown promise (Li et al., 2022; Zhu et al., 2023), targeting NEDD4 offers a potential advantage through simultaneous inhibition of multiple pathways, including PI3K, EGFR, TGF-β, and NOTCH. This approach may provide a more effective anti-cancer activity than single pathway inhibition.

## 6 Conclusion

This review synthesizes current understanding of the multidimensional mechanisms through which the NEDD4 family influences BC progression. The dual roles of NEDD4 proteins in BC emerge from their diverse substrate interactions and the distinct types of ubiquitin modifications they catalyze. While the NEDD4 family presents promising therapeutic targets, comprehensive elucidation of their regulatory mechanisms and functions requires additional investigation. Furthermore, validation of NEDD4 proteins as prognostic markers for BC patients necessitates larger prospective and retrospective clinical studies.

## Glossary

AJ: adhesion junction
BC: breast cancer
CDX: cell derived xenograft
circRNA: circular RNA
CSCs: cancer stem cells
DFS: disease-free survival
DMFS: distant metastasis-free survival
DRFS: distant relapse-free survival
EMT: epithelial-mesenchymal transition
ERK: extracellular signal-regulated kinase
IGF1R: type 1 insulin-like growth factor receptor
IHC: immunohistochemistry
IR: insulin receptor
JAC1: JWA gene activating compound 1
KLF5: krüppel-like factor 5
LATS1/2: large tumor suppressor kinases 1 and 2
lncRNA: long-chain non-coding RNA
miRNA: microRNA
MST1/2: mammalian STE20-like kinases 1 and 2
NDRG1: n-myc downstream-regulated gene 1
NICD1: NOTCH1 intracellular structural domain
OS: overall survival
PDX: patient derived xenograft
PIPKIγi5: type I γ-phosphatidylinositol phosphate 5-kinase i5
POI: protein of interest
PROTAC: proteolysis targeting chimeric
PRRG4: proline-rich γ-carboxyglutamic acid protein 4
PTEN: phosphatase and tensin homolog
RFS: recurrence-free survival
SAV1: salvador homolog 1
TAZ: ww domain-containing transcription regulator 1
TNBC: triple-negative breast cancer
TRAF4: tumor necrosis factor receptor-associated factor 4
TRAIL: tumor necrosis factor-related apoptosis-inducing ligand
Ub: ubiquitin
UPS: ubiquitin-proteasome system
USF2: upstream stimulatory factor 2
WBP2: ww domain-binding protein 2
YAP: yes-associated protein 1

## References

[B2] Agyin J. K. Santhamma B. Nair H. B. Roy S. S. Tekmal R. R. (2009). BU-32: a novel proteasome inhibitor for breast cancer. Breast cancer Res. BCR 11, R74. 10.1186/bcr2411 19821999 PMC2790855

[B3] Allemani C. Matsuda T. Di Carlo V. Harewood R. Matz M. Nikšić M. (2018). Global surveillance of trends in cancer survival 2000-14 (CONCORD-3): analysis of individual records for 37 513 025 patients diagnosed with one of 18 cancers from 322 population-based registries in 71 countries. Lancet London, Engl. 391, 1023–1075. 10.1016/S0140-6736(17)33326-3 PMC5879496 29395269

[B4] Badouel C. McNeill H. (2011). SnapShot: the hippo signaling pathway. Cell 145, 484–484.e481. 10.1016/j.cell.2011.04.009 21529719

[B5] Bai Y. Yang C. Hu K. Elly C. Liu Y. C. (2004). Itch E3 ligase-mediated regulation of TGF-beta signaling by modulating smad2 phosphorylation. Mol. Cell 15, 825–831. 10.1016/j.molcel.2004.07.021 15350225

[B6] Beňačka R. Szabóová D. Guľašová Z. Hertelyová Z. (2024). Non-coding RNAs in breast cancer: diagnostic and therapeutic implications. Int. J. Mol. Sci. 26, 127. 10.3390/ijms26010127 39795985 PMC11719911

[B7] Caballero A. Mahn S. A. Ali M. S. Rogers M. R. Marchese A. (2019). Heterologous regulation of CXCR4 lysosomal trafficking. J. Biol. Chem. 294, 8023–8036. 10.1074/jbc.RA118.005991 30936203 PMC6527173

[B1] Cancer Genome Atlas Network (2012). Comprehensive molecular portraits of human breast tumours. Nature 490, 61–70. 10.1038/nature11412 23000897 PMC3465532

[B8] Cao X. R. Lill N. L. Boase N. Shi P. P. Croucher D. R. Shan H. (2008). Nedd4 controls animal growth by regulating IGF-1 signaling. Sci. Signal. 1, ra5. 10.1126/scisignal.1160940 18812566 PMC2833362

[B9] Chan S. W. Lim C. J. Huang C. Chong Y. F. Gunaratne H. J. Hogue K. A. (2011). WW domain-mediated interaction with Wbp2 is important for the oncogenic property of TAZ. Oncogene 30, 600–610. 10.1038/onc.2010.438 20972459 PMC3033532

[B10] Chang L. Kamata H. Solinas G. Luo J. L. Maeda S. Venuprasad K. (2006). The E3 ubiquitin ligase itch couples JNK activation to TNFalpha-induced cell death by inducing c-FLIP(L) turnover. Cell 124, 601–613. 10.1016/j.cell.2006.01.021 16469705

[B11] Chang L. Shen L. Zhou H. Gao J. Pan H. Zheng L. (2019). ITCH nuclear translocation and H1.2 polyubiquitination negatively regulate the DNA damage response. Nucleic acids Res. 47, 824–842. 10.1093/nar/gky1199 30517763 PMC6344871

[B12] Chen C. Matesic L. E. (2007). The Nedd4-like family of E3 ubiquitin ligases and cancer. Cancer metastasis Rev. 26, 587–604. 10.1007/s10555-007-9091-x 17726579

[B13] Chen C. Sun X. Guo P. Dong X. Y. Sethi P. Cheng X. (2005). Human Kruppel-like factor 5 is a target of the E3 ubiquitin ligase WWP1 for proteolysis in epithelial cells. J. Biol. Chem. 280, 41553–41561. 10.1074/jbc.M506183200 16223724

[B14] Chen C. Zhou Z. Liu R. Li Y. Azmi P. B. Seth A. K. (2008). The WW domain containing E3 ubiquitin protein ligase 1 upregulates ErbB2 and EGFR through RING finger protein 11. Oncogene 27, 6845–6855. 10.1038/onc.2008.288 18724389

[B15] Chen C. Zhou Z. Ross J. S. Zhou W. Dong J. T. (2007). The amplified WWP1 gene is a potential molecular target in breast cancer. Int. J. cancer 121, 80–87. 10.1002/ijc.22653 17330240

[B16] Chen C. Zhou Z. Sheehan C. E. Slodkowska E. Sheehan C. B. Boguniewicz A. (2009). Overexpression of WWP1 is associated with the estrogen receptor and insulin-like growth factor receptor 1 in breast carcinoma. Int. J. cancer 124, 2829–2836. 10.1002/ijc.24266 19267401

[B17] Chen S. Wang H. Huang Y. F. Li M. L. Cheng J. H. Hu P. (2017). WW domain-binding protein 2: an adaptor protein closely linked to the development of breast cancer. Mol. cancer 16, 128. 10.1186/s12943-017-0693-9 28724435 PMC5518133

[B18] Choi S. Hedman A. C. Sayedyahossein S. Thapa N. Sacks D. B. Anderson R. A. (2016). Agonist-stimulated phosphatidylinositol-3,4,5-trisphosphate generation by scaffolded phosphoinositide kinases. Nat. Cell Biol. 18, 1324–1335. 10.1038/ncb3441 27870828 PMC5679705

[B19] Crudele F. Bianchi N. Reali E. Galasso M. Agnoletto C. Volinia S. (2020). The network of non-coding RNAs and their molecular targets in breast cancer. Mol. cancer 19, 61. 10.1186/s12943-020-01181-x 32188472 PMC7079433

[B20] David D. Jagadeeshan S. Hariharan R. Nair A. S. Pillai R. M. (2014). Smurf2 E3 ubiquitin ligase modulates proliferation and invasiveness of breast cancer cells in a CNKSR2 dependent manner. Cell Div. 9, 2. 10.1186/1747-1028-9-2 25191523 PMC4154384

[B21] David D. Surendran A. Thulaseedharan J. V. Nair A. S. (2018). Regulation of CNKSR2 protein stability by the HECT E3 ubiquitin ligase Smurf2, and its role in breast cancer progression. BMC cancer 18, 284. 10.1186/s12885-018-4188-x 29534682 PMC5850909

[B22] de Miguel D. Lemke J. Anel A. Walczak H. Martinez-Lostao L. (2016). Onto better TRAILs for cancer treatment. Cell death Differ. 23, 733–747. 10.1038/cdd.2015.174 26943322 PMC4832109

[B23] Deng Z. Fan T. Xiao C. Tian H. Zheng Y. Li C. (2024). TGF-β signaling in health, disease, and therapeutics. Signal Transduct. Target. Ther. 9, 61. 10.1038/s41392-024-01764-w 38514615 PMC10958066

[B24] Derynck R. Zhang Y. E. (2003). Smad-dependent and Smad-independent pathways in TGF-beta family signalling. Nature 425, 577–584. 10.1038/nature02006 14534577

[B25] Dey N. De P. Leyland-Jones B. (2017). PI3K-AKT-mTOR inhibitors in breast cancers: from tumor cell signaling to clinical trials. Pharmacol. and Ther. 175, 91–106. 10.1016/j.pharmthera.2017.02.037 28216025

[B26] Dittmar T. Husemann A. Schewe Y. Nofer J. R. Niggemann B. Zänker K. S. (2002). Induction of cancer cell migration by epidermal growth factor is initiated by specific phosphorylation of tyrosine 1248 of c-erbB-2 receptor via EGFR. FASEB J. 16, 1823–1825. 10.1096/fj.02-0096fje 12354693

[B27] Dixon S. J. Lemberg K. M. Lamprecht M. R. Skouta R. Zaitsev E. M. Gleason C. E. (2012). Ferroptosis: an iron-dependent form of nonapoptotic cell death. Cell 149, 1060–1072. 10.1016/j.cell.2012.03.042 22632970 PMC3367386

[B28] Ebisawa T. Fukuchi M. Murakami G. Chiba T. Tanaka K. Imamura T. (2001). Smurf1 interacts with transforming growth factor-beta type I receptor through Smad7 and induces receptor degradation. J. Biol. Chem. 276, 12477–12480. 10.1074/jbc.C100008200 11278251

[B29] El-Nassan H. B. (2013). Advances in the discovery of kinesin spindle protein (Eg5) inhibitors as antitumor agents. Eur. J. Med. Chem. 62, 614–631. 10.1016/j.ejmech.2013.01.031 23434636

[B30] Fan C. Wang Q. Kuipers T. B. Cats D. Iyengar P. V. Hagenaars S. C. (2023). LncRNA LITATS1 suppresses TGF-β-induced EMT and cancer cell plasticity by potentiating TβRI degradation. EMBO J. 42, e112806. 10.15252/embj.2022112806 36994542 PMC10183827

[B31] Feng S. M. Muraoka-Cook R. S. Hunter D. Sandahl M. A. Caskey L. S. Miyazawa K. (2009). The E3 ubiquitin ligase WWP1 selectively targets HER4 and its proteolytically derived signaling isoforms for degradation. Mol. Cell. Biol. 29, 892–906. 10.1128/MCB.00595-08 19047365 PMC2630679

[B32] Foroni C. Broggini M. Generali D. Damia G. (2012). Epithelial-mesenchymal transition and breast cancer: role, molecular mechanisms and clinical impact. Cancer Treat. Rev. 38, 689–697. 10.1016/j.ctrv.2011.11.001 22118888

[B33] Fouladkou F. Landry T. Kawabe H. Neeb A. Lu C. Brose N. (2008). The ubiquitin ligase Nedd4-1 is dispensable for the regulation of PTEN stability and localization. Proc. Natl. Acad. Sci. U. S. A. 105, 8585–8590. 10.1073/pnas.0803233105 18562292 PMC2438405

[B34] Fresno Vara J. A. Casado E. de Castro J. Cejas P. Belda-Iniesta C. González-Barón M. (2004). PI3K/Akt signalling pathway and cancer. Cancer Treat. Rev. 30, 193–204. 10.1016/j.ctrv.2003.07.007 15023437

[B35] Fu M. Hu Y. Lan T. Guan K. L. Luo T. Luo M. (2022). The Hippo signalling pathway and its implications in human health and diseases. Signal Transduct. Target. Ther. 7, 376. 10.1038/s41392-022-01191-9 36347846 PMC9643504

[B36] Fukunaga E. Inoue Y. Komiya S. Horiguchi K. Goto K. Saitoh M. (2008). Smurf2 induces ubiquitin-dependent degradation of Smurf1 to prevent migration of breast cancer cells. J. Biol. Chem. 283, 35660–35667. 10.1074/jbc.M710496200 18927080

[B37] Gallagher E. J. Fei K. Feldman S. M. Port E. Friedman N. B. Boolbol S. K. (2020). Insulin resistance contributes to racial disparities in breast cancer prognosis in US women. Breast cancer Res. BCR 22, 40. 10.1186/s13058-020-01281-y 32393319 PMC7216707

[B38] Ge M. K. Zhang N. Xia L. Zhang C. Dong S. S. Li Z. M. (2020). FBXO22 degrades nuclear PTEN to promote tumorigenesis. Nat. Commun. 11, 1720. 10.1038/s41467-020-15578-1 32249768 PMC7136256

[B39] Giaquinto A. N. Sung H. Miller K. D. Kramer J. L. Newman L. A. Minihan A. (2022). Breast cancer statistics, 2022. CA a cancer J. Clin. 72, 524–541. 10.3322/caac.21754 36190501

[B40] Gough S. M. Flanagan J. J. Teh J. Andreoli M. Rousseau E. Pannone M. (2024). Oral estrogen receptor PROTAC vepdegestrant (ARV-471) is highly efficacious as monotherapy and in combination with CDK4/6 or PI3K/mTOR pathway inhibitors in preclinical ER+ breast cancer models. Clin. cancer Res. official J. Am. Assoc. Cancer Res. 30, 3549–3563. 10.1158/1078-0432.CCR-23-3465 PMC11325148 38819400

[B41] Grönroos E. Hellman U. Heldin C. H. Ericsson J. (2002). Control of Smad7 stability by competition between acetylation and ubiquitination. Mol. Cell 10, 483–493. 10.1016/s1097-2765(02)00639-1 12408818

[B42] Gu Y. Gao H. Zhang H. John A. Zhu X. Shivaram S. (2022). TRAF4 hyperactivates HER2 signaling and contributes to Trastuzumab resistance in HER2-positive breast cancer. Oncogene 41, 4119–4129. 10.1038/s41388-022-02415-6 35864174 PMC9417995

[B43] Guarnieri A. L. Towers C. G. Drasin D. J. Oliphant M. U. J. Andrysik Z. Hotz T. J. (2018). The miR-106b-25 cluster mediates breast tumor initiation through activation of NOTCH1 via direct repression of NEDD4L. Oncogene 37, 3879–3893. 10.1038/s41388-018-0239-7 29662198 PMC6043359

[B44] Guerrero-Zotano A. Mayer I. A. Arteaga C. L. (2016). PI3K/AKT/mTOR: role in breast cancer progression, drug resistance, and treatment. Cancer metastasis Rev. 35, 515–524. 10.1007/s10555-016-9637-x 27896521

[B45] Gumbiner B. M. (2005). Regulation of cadherin-mediated adhesion in morphogenesis. Nat. Rev. Mol. Cell Biol. 6, 622–634. 10.1038/nrm1699 16025097

[B46] Guo J. Cheng J. Zheng N. Zhang X. Dai X. Zhang L. (2021). Copper promotes tumorigenesis by activating the PDK1-AKT oncogenic pathway in a copper transporter 1 dependent manner. Adv. Sci. Weinheim, Baden-Wurttemberg, Ger. 8, e2004303. 10.1002/advs.202004303 PMC8456201 34278744

[B47] Guo X. Y. Liu T. T. Zhu W. J. Liu H. T. Zhang G. H. Song L. (2022). CircKDM4B suppresses breast cancer progression via the miR-675/NEDD4L axis. Oncogene 41, 1895–1906. 10.1038/s41388-022-02232-x 35145234

[B48] Guo Y. Cui Y. Li Y. Jin X. Wang D. Lei M. (2023). Cytoplasmic YAP1-mediated ESCRT-III assembly promotes autophagic cell death and is ubiquitinated by NEDD4L in breast cancer. Cancer Commun. Lond. Engl. 43, 582–612. 10.1002/cac2.12417 PMC10174091 37005481

[B49] Haag C. Stadel D. Zhou S. Bachem M. G. Möller P. Debatin K. M. (2011). Identification of c-FLIP(L) and c-FLIP(S) as critical regulators of death receptor-induced apoptosis in pancreatic cancer cells. Gut 60, 225–237. 10.1136/gut.2009.202325 20876774

[B50] Hamilton E. P. Ma C. De Laurentiis M. Iwata H. Hurvitz S. A. Wander S. A. (2024). VERITAC-2: a Phase III study of vepdegestrant, a PROTAC ER degrader, versus fulvestrant in ER+/HER2-advanced breast cancer. Future Oncol. Lond. Engl. 20, 2447–2455. 10.1080/14796694.2024.2377530 PMC11524203 39072356

[B51] Hao M. Zhang J. Sun M. Diao K. Wang J. Li S. (2022). TRAF4 inhibits the apoptosis and promotes the proliferation of breast cancer cells by inhibiting the ubiquitination of spindle assembly-associated protein Eg5. Front. Oncol. 12, 855139. 10.3389/fonc.2022.855139 35692762 PMC9174544

[B52] Harvey K. F. Kumar S. (1999). Nedd4-like proteins: an emerging family of ubiquitin-protein ligases implicated in diverse cellular functions. Trends Cell Biol. 9, 166–169. 10.1016/s0962-8924(99)01541-x 10322449

[B53] Hershko A. Heller H. Elias S. Ciechanover A. (1983). Components of ubiquitin-protein ligase system. Resolution, affinity purification, and role in protein breakdown. J. Biol. Chem. 258, 8206–8214. 10.1016/s0021-9258(20)82050-x 6305978

[B54] Ho K. C. Zhou Z. She Y. M. Chun A. Cyr T. D. Yang X. (2011). Itch E3 ubiquitin ligase regulates large tumor suppressor 1 stability [corrected]. Proc. Natl. Acad. Sci. U. S. A. 108, 4870–4875. 10.1073/pnas.1101273108 21383157 PMC3064387

[B55] Hong S. W. Moon J. H. Kim J. S. Shin J. S. Jung K. A. Lee W. K. (2014). p34 is a novel regulator of the oncogenic behavior of NEDD4-1 and PTEN. Cell death Differ. 21, 146–160. 10.1038/cdd.2013.141 24141722 PMC3857621

[B56] Hsu J. L. Hung M. C. (2016). The role of HER2, EGFR, and other receptor tyrosine kinases in breast cancer. Cancer metastasis Rev. 35, 575–588. 10.1007/s10555-016-9649-6 27913999 PMC5215954

[B57] Huang Q. Zhang R. Xia Y. Shen J. Dong H. Li X. (2023). DAB2IP suppresses invadopodia formation through destabilizing ALK by interacting with USP10 in breast cancer. iScience 26, 107606. 10.1016/j.isci.2023.107606 37664607 PMC10470318

[B58] Huang Z. Choi B. K. Mujoo K. Fan X. Fa M. Mukherjee S. (2015). The E3 ubiquitin ligase NEDD4 negatively regulates HER3/ErbB3 level and signaling. Oncogene 34, 1105–1115. 10.1038/onc.2014.56 24662824

[B59] Imamura T. Hikita A. Inoue Y. (2012). The roles of TGF-β signaling in carcinogenesis and breast cancer metastasis. Breast cancer Tokyo, Jpn. 19, 118–124. 10.1007/s12282-011-0321-2 22139728

[B60] Ingham R. J. Gish G. Pawson T. (2004). The Nedd4 family of E3 ubiquitin ligases: functional diversity within a common modular architecture. Oncogene 23, 1972–1984. 10.1038/sj.onc.1207436 15021885

[B61] Ito I. Hanyu A. Wayama M. Goto N. Katsuno Y. Kawasaki S. (2010). Estrogen inhibits transforming growth factor beta signaling by promoting Smad2/3 degradation. J. Biol. Chem. 285, 14747–14755. 10.1074/jbc.M109.093039 20207742 PMC2863224

[B62] Iwata H. Naito Y. Hattori M. Yoshimura A. Yonemori K. Aizawa M. (2025). Safety and pharmacokinetics of vepdegestrant in Japanese patients with ER+ advanced breast cancer: a phase 1 study. Int. J. Clin. Oncol. 30, 72–82. 10.1007/s10147-024-02648-3 39565495 PMC11700046

[B63] Jamdade V. S. Sethi N. Mundhe N. A. Kumar P. Lahkar M. Sinha N. (2015). Therapeutic targets of triple-negative breast cancer: a review. Br. J. Pharmacol. 172, 4228–4237. 10.1111/bph.13211 26040571 PMC4556464

[B64] Jeon S. A. Kim D. W. Lee D. B. Cho J. Y. (2020). NEDD4 plays roles in the maintenance of breast cancer stem cell characteristics. Front. Oncol. 10, 1680. 10.3389/fonc.2020.01680 33014839 PMC7509455

[B65] Jia L. Zhou Z. Liang H. Wu J. Shi P. Li F. (2016). KLF5 promotes breast cancer proliferation, migration and invasion in part by upregulating the transcription of TNFAIP2. Oncogene 35, 2040–2051. 10.1038/onc.2015.263 26189798

[B66] Jin C. Yang Y. A. Anver M. R. Morris N. Wang X. Zhang Y. E. (2009). Smad ubiquitination regulatory factor 2 promotes metastasis of breast cancer cells by enhancing migration and invasiveness. Cancer Res. 69, 735–740. 10.1158/0008-5472.CAN-08-1463 19155312 PMC2639752

[B67] Jung S. Li C. Jeong D. Lee S. Ohk J. Park M. (2013). Oncogenic function of p34SEI-1 via NEDD4-1-mediated PTEN ubiquitination/degradation and activation of the PI3K/AKT pathway. Int. J. Oncol. 43, 1587–1595. 10.3892/ijo.2013.2064 23970032

[B68] Kavsak P. Rasmussen R. K. Causing C. G. Bonni S. Zhu H. Thomsen G. H. (2000). Smad7 binds to Smurf2 to form an E3 ubiquitin ligase that targets the TGF beta receptor for degradation. Mol. Cell 6, 1365–1375. 10.1016/s1097-2765(00)00134-9 11163210

[B69] Kern J. G. Tilston-Lunel A. M. Federico A. Ning B. Mueller A. Peppler G. B. (2022). Inactivation of LATS1/2 drives luminal-basal plasticity to initiate basal-like mammary carcinomas. Nat. Commun. 13, 7198. 10.1038/s41467-022-34864-8 36443313 PMC9705439

[B70] Kishikawa T. Higuchi H. Wang L. Panch N. Maymi V. Best S. (2021). WWP1 inactivation enhances efficacy of PI3K inhibitors while suppressing their toxicities in breast cancer models. J. Clin. investigation 131, e140436. 10.1172/JCI140436 PMC8670846 34907909

[B71] Komuro A. Imamura T. Saitoh M. Yoshida Y. Yamori T. Miyazono K. (2004). Negative regulation of transforming growth factor-beta (TGF-beta) signaling by WW domain-containing protein 1 (WWP1). Oncogene 23, 6914–6923. 10.1038/sj.onc.1207885 15221015

[B72] Kotb R. M. Ibrahim S. S. Mostafa O. M. Shahin N. N. (2022). Potential role of CXCR4 in trastuzumab resistance in breast cancer patients. Mol. basis Dis. 1868, 166520. 10.1016/j.bbadis.2022.166520 35985446

[B73] Krishna B. M. Jana S. Singhal J. Horne D. Awasthi S. Salgia R. (2019). Notch signaling in breast cancer: from pathway analysis to therapy. Cancer Lett. 461, 123–131. 10.1016/j.canlet.2019.07.012 31326555 PMC9003668

[B74] Kuratomi G. Komuro A. Goto K. Shinozaki M. Miyazawa K. Miyazono K. (2005). NEDD4-2 (neural precursor cell expressed, developmentally down-regulated 4-2) negatively regulates TGF-beta (transforming growth factor-beta) signalling by inducing ubiquitin-mediated degradation of Smad2 and TGF-beta type I receptor. Biochem. J. 386, 461–470. 10.1042/BJ20040738 15496141 PMC1134864

[B75] Kwon A. Lee H. L. Woo K. M. Ryoo H. M. Baek J. H. (2013). SMURF1 plays a role in EGF-induced breast cancer cell migration and invasion. Mol. cells 36, 548–555. 10.1007/s10059-013-0233-4 24241683 PMC3887964

[B76] Lee Y. R. Chen M. Lee J. D. Zhang J. Lin S. Y. Fu T. M. (2019). Reactivation of PTEN tumor suppressor for cancer treatment through inhibition of a MYC-WWP1 inhibitory pathway. Science 364, eaau0159. 10.1126/science.aau0159 31097636 PMC7081834

[B77] Lei G. Zhang Y. Koppula P. Liu X. Zhang J. Lin S. H. (2020). The role of ferroptosis in ionizing radiation-induced cell death and tumor suppression. Cell Res. 30, 146–162. 10.1038/s41422-019-0263-3 31949285 PMC7015061

[B78] Li J. Wang P. Xie Z. Wang S. Cen S. Li M. (2019). TRAF4 positively regulates the osteogenic differentiation of mesenchymal stem cells by acting as an E3 ubiquitin ligase to degrade Smurf2. Cell death Differ. 26, 2652–2666. 10.1038/s41418-019-0328-3 31076633 PMC7224386

[B79] Li L. Guturi K. K. N. Gautreau B. Patel P. S. Saad A. Morii M. (2018). Ubiquitin ligase RNF8 suppresses Notch signaling to regulate mammary development and tumorigenesis. J. Clin. investigation 128, 4525–4542. 10.1172/JCI120401 PMC6160000 30222135

[B80] Li S. Lu K. Wang J. An L. Yang G. Chen H. (2010). Ubiquitin ligase Smurf1 targets TRAF family proteins for ubiquitination and degradation. Mol. Cell. Biochem. 338, 11–17. 10.1007/s11010-009-0315-y 19937093

[B81] Li X. Dai X. Wan L. Inuzuka H. Sun L. North B. J. (2016). Smurf1 regulation of DAB2IP controls cell proliferation and migration. Oncotarget 7, 26057–26069. 10.18632/oncotarget.8424 27036023 PMC5041964

[B82] Li Y. Li W. Ying Z. Tian H. Zhu X. Li J. (2014). Metastatic heterogeneity of breast cancer cells is associated with expression of a heterogeneous TGFβ-activating miR424-503 gene cluster. Cancer Res. 74, 6107–6118. 10.1158/0008-5472.CAN-14-0389 25164015

[B83] Li Y. Zhang H. Merkher Y. Chen L. Liu N. Leonov S. (2022). Recent advances in therapeutic strategies for triple-negative breast cancer. J. Hematol. and Oncol. 15, 121. 10.1186/s13045-022-01341-0 36038913 PMC9422136

[B84] Li Y. Zhou Z. Alimandi M. Chen C. (2009). WW domain containing E3 ubiquitin protein ligase 1 targets the full-length ErbB4 for ubiquitin-mediated degradation in breast cancer. Oncogene 28, 2948–2958. 10.1038/onc.2009.162 19561640

[B85] Li Y. M. Pan Y. Wei Y. Cheng X. Zhou B. P. Tan M. (2004). Upregulation of CXCR4 is essential for HER2-mediated tumor metastasis. Cancer Cell 6, 459–469. 10.1016/j.ccr.2004.09.027 15542430

[B86] Lim S. K. Lu S. Y. Kang S. A. Tan H. J. Li Z. Adrian Wee Z. N. (2016). Wnt signaling promotes breast cancer by blocking ITCH-mediated degradation of YAP/TAZ transcriptional coactivator WBP2. Cancer Res. 76, 6278–6289. 10.1158/0008-5472.CAN-15-3537 27578003

[B87] Lin X. Liang M. Feng X. H. (2000). Smurf2 is a ubiquitin E3 ligase mediating proteasome-dependent degradation of Smad2 in transforming growth factor-beta signaling. J. Biol. Chem. 275, 36818–36822. 10.1074/jbc.C000580200 11016919

[B88] Liu J. Wan L. Liu J. Yuan Z. Zhang J. Guo J. (2016). Cdh1 inhibits WWP2-mediated ubiquitination of PTEN to suppress tumorigenesis in an APC-independent manner. Cell Discov. 2, 15044. 10.1038/celldisc.2015.44 27462441 PMC4860961

[B89] Liu J. Wan L. Liu P. Inuzuka H. Liu J. Wang Z. (2014). SCF(β-TRCP)-mediated degradation of NEDD4 inhibits tumorigenesis through modulating the PTEN/Akt signaling pathway. Oncotarget 5, 1026–1037. 10.18632/oncotarget.1675 24657926 PMC4011580

[B90] Liu L. Zhang C. Qu S. Liu R. Chen H. Liang Z. (2022b). ESR1 inhibits ionizing radiation-induced ferroptosis in breast cancer cells via the NEDD4L/CD71 pathway. Archives Biochem. biophysics 725, 109299. 10.1016/j.abb.2022.109299 35613689

[B91] Liu R. Liu L. Bian Y. Zhang S. Wang Y. Chen H. (2021). The dual regulation effects of ESR1/nedd4l on SLC7A11 in breast cancer under ionizing radiation. Front. Cell Dev. Biol. 9, 772380. 10.3389/fcell.2021.772380 35252218 PMC8888677

[B92] Liu Z. Meng D. Wang J. Cao H. Feng P. Wu S. (2022a). GASP1 enhances malignant phenotypes of breast cancer cells and decreases their response to paclitaxel by forming a vicious cycle with IGF1/IGF1R signaling pathway. Cell death and Dis. 13, 751. 10.1038/s41419-022-05198-6 PMC9427794 36042202

[B93] Lucas L. M. Dwivedi V. Senfeld J. I. Cullum R. L. Mill C. P. Piazza J. T. (2022). The yin and Yang of ERBB4: tumor suppressor and oncoprotein. Pharmacol. Rev. 74, 18–47. 10.1124/pharmrev.121.000381 34987087 PMC11060329

[B94] Luhtala S. Staff S. Kallioniemi A. Tanner M. Isola J. (2018). Clinicopathological and prognostic correlations of HER3 expression and its degradation regulators, NEDD4-1 and NRDP1, in primary breast cancer. BMC cancer 18, 1045. 10.1186/s12885-018-4917-1 30367623 PMC6204010

[B95] Luo M. Li J. Yang Q. Xu S. Zhang K. Chen J. (2022). N4BP3 promotes breast cancer metastasis via NEDD4-mediated E-cadherin ubiquitination and degradation. Cancer Lett. 550, 215926. 10.1016/j.canlet.2022.215926 36162713

[B96] Ma S. Meng Z. Chen R. Guan K. L. (2019). The hippo pathway: biology and pathophysiology. Annu. Rev. Biochem. 88, 577–604. 10.1146/annurev-biochem-013118-111829 30566373

[B97] Marchese A. Raiborg C. Santini F. Keen J. H. Stenmark H. Benovic J. L. (2003). The E3 ubiquitin ligase AIP4 mediates ubiquitination and sorting of the G protein-coupled receptor CXCR4. Dev. Cell 5, 709–722. 10.1016/s1534-5807(03)00321-6 14602072

[B98] Marcu L. G. (2020). Imaging biomarkers of tumour proliferation and invasion for personalised lung cancer therapy. J. personalized Med. 10, 222. 10.3390/jpm10040222 PMC7711676 33198090

[B99] Massagué J. (2000). How cells read TGF-beta signals. Nat. Rev. Mol. Cell Biol. 1, 169–178. 10.1038/35043051 11252892

[B100] Mayayo-Peralta I. Prekovic S. Zwart W. (2021). Estrogen Receptor on the move: cistromic plasticity and its implications in breast cancer. Mol. aspects Med. 78, 100939. 10.1016/j.mam.2020.100939 33358533

[B101] Mayer I. A. Abramson V. G. Lehmann B. D. Pietenpol J. A. (2014). New strategies for triple-negative breast cancer--deciphering the heterogeneity. Clin. cancer Res. official J. Am. Assoc. Cancer Res. 20, 782–790. 10.1158/1078-0432.CCR-13-0583 PMC3962777 24536073

[B102] Miyazawa K. Miyazono K. (2017). Regulation of TGF-β family signaling by inhibitory smads. Cold Spring Harb. Perspect. Biol. 9, a022095. 10.1101/cshperspect.a022095 27920040 PMC5334261

[B103] Morén A. Imamura T. Miyazono K. Heldin C. H. Moustakas A. (2005). Degradation of the tumor suppressor Smad4 by WW and HECT domain ubiquitin ligases. J. Biol. Chem. 280, 22115–22123. 10.1074/jbc.M414027200 15817471

[B104] Müller A. Homey B. Soto H. Ge N. Catron D. Buchanan M. E. (2001). Involvement of chemokine receptors in breast cancer metastasis. Nature 410, 50–56. 10.1038/35065016 11242036

[B105] Natori Y. Suga J. Tokuda E. Tachibana K. Imai J. I. Honma R. (2023). E3 ubiquitin ligase NEDD4 affects estrogen receptor α expression and the prognosis of patients with hormone receptor-positive breast cancer. Cancers 15, 539. 10.3390/cancers15020539 36672488 PMC9857178

[B106] Neklesa T. K. Winkler J. D. Crews C. M. (2017). Targeted protein degradation by PROTACs. Pharmacol. and Ther. 174, 138–144. 10.1016/j.pharmthera.2017.02.027 28223226

[B107] Nguyen T. N. Q. Jung S. Nguyen H. A. Lee B. Vu S. H. Myagmarjav D. (2022). The regulation of insulin receptor/insulin-like growth factor 1 receptor ratio, an important factor for breast cancer prognosis, by TRIP-Br1. J. Hematol. and Oncol. 15, 82. 10.1186/s13045-022-01303-6 35710446 PMC9204904

[B108] Nguyen Huu N. S. Ryder W. D. Zeps N. Flasza M. Chiu M. Hanby A. M. (2008). Tumour-promoting activity of altered WWP1 expression in breast cancer and its utility as a prognostic indicator. J. pathology 216, 93–102. 10.1002/path.2385 18604872

[B109] Nunnery S. E. Mayer I. A. (2020). Targeting the PI3K/AKT/mTOR pathway in hormone-positive breast cancer. Drugs 80, 1685–1697. 10.1007/s40265-020-01394-w 32894420 PMC7572750

[B110] Padgett R. W. Cho S. H. Evangelista C. (1998). Smads are the central component in transforming growth factor-beta signaling. Pharmacol. and Ther. 78, 47–52. 10.1016/s0163-7258(97)00166-6 9593329

[B111] Piggott L. Silva A. Robinson T. Santiago-Gómez A. Simões B. M. Becker M. (2018). Acquired resistance of ER-positive breast cancer to endocrine treatment confers an adaptive sensitivity to TRAIL through posttranslational downregulation of c-FLIP. Clin. cancer Res. official J. Am. Assoc. Cancer Res. 24, 2452–2463. 10.1158/1078-0432.CCR-17-1381 29363524

[B112] Ren Y. Chen D. Zhai Z. Chen J. Li A. Liang Y. (2021). JAC1 suppresses proliferation of breast cancer through the JWA/p38/SMURF1/HER2 signaling. Cell death Discov. 7, 85. 10.1038/s41420-021-00426-y 33875644 PMC8055679

[B113] Rossi M. Rotblat B. Ansell K. Amelio I. Caraglia M. Misso G. (2014). High throughput screening for inhibitors of the HECT ubiquitin E3 ligase ITCH identifies antidepressant drugs as regulators of autophagy. Cell death and Dis. 5, e1203. 10.1038/cddis.2014.113 PMC4047876 24787015

[B114] Rotin D. Kumar S. (2009). Physiological functions of the HECT family of ubiquitin ligases. Nat. Rev. Mol. Cell Biol. 10, 398–409. 10.1038/nrm2690 19436320

[B115] Rotin D. Prag G. (2024). Physiological functions of the ubiquitin ligases nedd4-1 and nedd4-2. Physiol. Bethesda, Md 39, 18–29. 10.1152/physiol.00023.2023 37962894

[B116] Sahai E. Garcia-Medina R. Pouysségur J. Vial E. (2007). Smurf1 regulates tumor cell plasticity and motility through degradation of RhoA leading to localized inhibition of contractility. J. Cell Biol. 176, 35–42. 10.1083/jcb.200605135 17190792 PMC2063621

[B117] Salah Z. Melino G. Aqeilan R. I. (2011). Negative regulation of the Hippo pathway by E3 ubiquitin ligase ITCH is sufficient to promote tumorigenicity. Cancer Res. 71, 2010–2020. 10.1158/0008-5472.CAN-10-3516 21212414

[B118] Schmid P. Kühnhardt D. Kiewe P. Lehenbauer-Dehm S. Schippinger W. Greil R. (2008). A phase I/II study of bortezomib and capecitabine in patients with metastatic breast cancer previously treated with taxanes and/or anthracyclines. Ann. Oncol. official J. Eur. Soc. Med. Oncol. 19, 871–876. 10.1093/annonc/mdm569 18209010

[B119] Schroeter E. H. Kisslinger J. A. Kopan R. (1998). Notch-1 signalling requires ligand-induced proteolytic release of intracellular domain. Nature 393, 382–386. 10.1038/30756 9620803

[B120] Schwartz A. L. Ciechanover A. (1999). The ubiquitin-proteasome pathway and pathogenesis of human diseases. Annu. Rev. Med. 50, 57–74. 10.1146/annurev.med.50.1.57 10073263

[B121] Schwartz G. Shee K. Romo B. Marotti J. Kisselev A. Lewis L. (2021). Phase ib study of the oral proteasome inhibitor ixazomib (MLN9708) and fulvestrant in advanced ER+ breast cancer progressing on fulvestrant. Oncol. 26, 467–e924. 10.1002/onco.13733 PMC8176977 33641211

[B122] Seo S. R. Lallemand F. Ferrand N. Pessah M. L'Hoste S. Camonis J. (2004). The novel E3 ubiquitin ligase Tiul1 associates with TGIF to target Smad2 for degradation. EMBO J. 23, 3780–3792. 10.1038/sj.emboj.7600398 15359284 PMC522797

[B123] Serwe G. Kachaner D. Gagnon J. Plutoni C. Lajoie D. Duramé E. (2023). CNK2 promotes cancer cell motility by mediating ARF6 activation downstream of AXL signalling. Nat. Commun. 14, 3560. 10.1038/s41467-023-39281-z 37322019 PMC10272126

[B124] Sharma V. Sharma A. K. Punj V. Priya P. (2019). Recent nanotechnological interventions targeting PI3K/Akt/mTOR pathway: a focus on breast cancer. Seminars cancer Biol. 59, 133–146. 10.1016/j.semcancer.2019.08.005 31408722

[B125] Shen S. Du X. J. Liu J. Sun R. Zhu Y. H. Wang J. (2015). Delivery of bortezomib with nanoparticles for basal-like triple-negative breast cancer therapy. J. Control. release official J. Control. Release Soc. 208, 14–24. 10.1016/j.jconrel.2014.12.043 25575864

[B126] Shin S. Kim K. Kim H. R. Ylaya K. Do S. I. Hewitt S. M. (2020). Deubiquitylation and stabilization of Notch1 intracellular domain by ubiquitin-specific protease 8 enhance tumorigenesis in breast cancer. Cell death Differ. 27, 1341–1354. 10.1038/s41418-019-0419-1 31527799 PMC7206187

[B127] Siegel R. L. Giaquinto A. N. Jemal A. (2024). Cancer statistics. CA a cancer J. Clin. 74, 12–49. 10.3322/caac.21820 38230766

[B128] Simmons M. J. Serra R. Hermance N. Kelliher M. A. (2012). NOTCH1 inhibition *in vivo* results in mammary tumor regression and reduced mammary tumorsphere-forming activity *in vitro* . Breast cancer Res. BCR 14, R126. 10.1186/bcr3321 22992387 PMC4053103

[B129] Stambolic V. Suzuki A. de la Pompa J. L. Brothers G. M. Mirtsos C. Sasaki T. (1998). Negative regulation of PKB/Akt-dependent cell survival by the tumor suppressor PTEN. Cell 95, 29–39. 10.1016/s0092-8674(00)81780-8 9778245

[B130] Subik K. Shu L. Wu C. Liang Q. Hicks D. Boyce B. (2012). The ubiquitin E3 ligase WWP1 decreases CXCL12-mediated MDA231 breast cancer cell migration and bone metastasis. Bone 50, 813–823. 10.1016/j.bone.2011.12.022 22266093 PMC3439807

[B131] Suga J. Izumiyama K. Tanaka N. Saji S. (2018). Estradiol promotes rapid degradation of HER3 in ER-positive breast cancer cell line MCF-7. Biochem. biophysics Rep. 16, 103–109. 10.1016/j.bbrep.2018.10.008 PMC6205365 30417127

[B132] Sun M. Cai J. Anderson R. A. Sun Y. (2016). Type I γ phosphatidylinositol phosphate 5-kinase i5 controls the ubiquitination and degradation of the tumor suppressor mitogen-inducible gene 6. J. Biol. Chem. 291, 21461–21473. 10.1074/jbc.M116.736041 27557663 PMC5076818

[B133] Swatek K. N. Komander D. (2016). Ubiquitin modifications. Cell Res. 26, 399–422. 10.1038/cr.2016.39 27012465 PMC4822133

[B134] Tan Y. Chen Y. Du M. Peng Z. Xie P. (2019). USF2 inhibits the transcriptional activity of Smurf1 and Smurf2 to promote breast cancer tumorigenesis. Cell. Signal. 53, 49–58. 10.1016/j.cellsig.2018.09.013 30244169

[B135] Tao J. J. Castel P. Radosevic-Robin N. Elkabets M. Auricchio N. Aceto N. (2014). Antagonism of EGFR and HER3 enhances the response to inhibitors of the PI3K-Akt pathway in triple-negative breast cancer. Sci. Signal. 7, ra29. 10.1126/scisignal.2005125 24667376 PMC4283215

[B136] Tong D. Czerwenka K. Heinze G. Ryffel M. Schuster E. Witt A. (2006). Expression of KLF5 is a prognostic factor for disease-free survival and overall survival in patients with breast cancer. Clin. cancer Res. official J. Am. Assoc. Cancer Res. 12, 2442–2448. 10.1158/1078-0432.CCR-05-0964 16638850

[B137] Tran M. H. Seo E. Min S. Nguyen Q. T. Choi J. Lee U. J. (2018). NEDD4-induced degradative ubiquitination of phosphatidylinositol 4-phosphate 5-kinase α and its implication in breast cancer cell proliferation. J. Cell. Mol. Med. 22, 4117–4129. 10.1111/jcmm.13689 29851245 PMC6111810

[B138] Trotman L. C. Wang X. Alimonti A. Chen Z. Teruya-Feldstein J. Yang H. (2007). Ubiquitination regulates PTEN nuclear import and tumor suppression. Cell 128, 141–156. 10.1016/j.cell.2006.11.040 17218261 PMC1855245

[B139] Ulanet D. B. Ludwig D. L. Kahn C. R. Hanahan D. (2010). Insulin receptor functionally enhances multistage tumor progression and conveys intrinsic resistance to IGF-1R targeted therapy. Proc. Natl. Acad. Sci. U. S. A. 107, 10791–10798. 10.1073/pnas.0914076107 20457905 PMC2890766

[B140] Veikkolainen V. Vaparanta K. Halkilahti K. Iljin K. Sundvall M. Elenius K. (2011). Function of ERBB4 is determined by alternative splicing. Cell cycleGeorget. Tex. 10, 2647–2657. 10.4161/cc.10.16.17194 21811097

[B141] Verma N. Müller A. K. Kothari C. Panayotopoulou E. Kedan A. Selitrennik M. (2017). Targeting of PYK2 synergizes with EGFR antagonists in basal-like TNBC and circumvents HER3-associated resistance via the NEDD4-NDRG1 Axis. Cancer Res. 77, 86–99. 10.1158/0008-5472.CAN-16-1797 27793840

[B142] Wali V. B. Haskins J. W. Gilmore-Hebert M. Platt J. T. Liu Z. Stern D. F. (2014). Convergent and divergent cellular responses by ErbB4 isoforms in mammary epithelial cells. Mol. cancer Res. MCR 12, 1140–1155. 10.1158/1541-7786.MCR-13-0637 24829397 PMC4728083

[B143] Wan L. Liu T. Hong Z. Pan Y. Sizemore S. T. Zhang J. (2019). NEDD4 expression is associated with breast cancer progression and is predictive of a poor prognosis. Breast cancer Res. BCR 21, 148. 10.1186/s13058-019-1236-7 31856858 PMC6923956

[B144] Wan L. Zou W. Gao D. Inuzuka H. Fukushima H. Berg A. H. (2011). Cdh1 regulates osteoblast function through an APC/C-independent modulation of Smurf1. Mol. Cell 44, 721–733. 10.1016/j.molcel.2011.09.024 22152476 PMC3240853

[B145] Wang H. Shi Y. Chen C. H. Wen Y. Zhou Z. Yang C. (2021a). KLF5-induced lncRNA IGFL2-AS1 promotes basal-like breast cancer cell growth and survival by upregulating the expression of IGFL1. Cancer Lett. 515, 49–62. 10.1016/j.canlet.2021.04.016 34052325

[B146] Wang H. R. Zhang Y. Ozdamar B. Ogunjimi A. A. Alexandrova E. Thomsen G. H. (2003). Regulation of cell polarity and protrusion formation by targeting RhoA for degradation. Sci. (New York, N.Y.) 302, 1775–1779. 10.1126/science.1090772 14657501

[B147] Wang L. Zhou Y. Jiang L. Lu L. Dai T. Li A. (2021b). CircWAC induces chemotherapeutic resistance in triple-negative breast cancer by targeting miR-142, upregulating WWP1 and activating the PI3K/AKT pathway. Mol. cancer 20, 43. 10.1186/s12943-021-01332-8 33648498 PMC7919093

[B148] Wang X. Jin C. Tang Y. Tang L. Y. Zhang Y. E. (2013). Ubiquitination of tumor necrosis factor receptor-associated factor 4 (TRAF4) by Smad ubiquitination regulatory factor 1 (Smurf1) regulates motility of breast epithelial and cancer cells. J. Biol. Chem. 288, 21784–21792. 10.1074/jbc.M113.472704 23760265 PMC3724635

[B149] Wang X. Trotman L. C. Koppie T. Alimonti A. Chen Z. Gao Z. (2007). NEDD4-1 is a proto-oncogenic ubiquitin ligase for PTEN. Cell 128, 129–139. 10.1016/j.cell.2006.11.039 17218260 PMC1828909

[B150] Wang Z. Dang T. Liu T. Chen S. Li L. Huang S. (2016). NEDD4L protein catalyzes ubiquitination of PIK3CA protein and regulates PI3K-AKT signaling. J. Biol. Chem. 291, 17467–17477. 10.1074/jbc.M116.726083 27339899 PMC5016142

[B151] Wang Z. Liu Z. Chen X. Li J. Yao W. Huang S. (2019). A multi-lock inhibitory mechanism for fine-tuning enzyme activities of the HECT family E3 ligases. Nat. Commun. 10, 3162. 10.1038/s41467-019-11224-7 31320636 PMC6639328

[B152] Wang Z. W. Hu X. Ye M. Lin M. Chu M. Shen X. (2020). NEDD4 E3 ligase: functions and mechanism in human cancer. Seminars cancer Biol. 67, 92–101. 10.1016/j.semcancer.2020.03.006 32171886

[B153] Wei W. Lewis M. T. (2015). Identifying and targeting tumor-initiating cells in the treatment of breast cancer. Endocrine-related cancer 22, R135–R155. 10.1530/ERC-14-0447 25876646 PMC4447610

[B154] Wiesner S. Ogunjimi A. A. Wang H. R. Rotin D. Sicheri F. Wrana J. L. (2007). Autoinhibition of the HECT-type ubiquitin ligase Smurf2 through its C2 domain. Cell 130, 651–662. 10.1016/j.cell.2007.06.050 17719543

[B155] Wu Q. Li G. Wen C. Zeng T. Fan Y. Liu C. (2020). Monoubiquitination of p120-catenin is essential for TGFβ-induced epithelial-mesenchymal transition and tumor metastasis. Sci. Adv. 6, eaay9819. 10.1126/sciadv.aay9819 32010791 PMC6976293

[B156] Xie Y. Avello M. Schirle M. McWhinnie E. Feng Y. Bric-Furlong E. (2013). Deubiquitinase FAM/USP9X interacts with the E3 ubiquitin ligase SMURF1 protein and protects it from ligase activity-dependent self-degradation. J. Biol. Chem. 288, 2976–2985. 10.1074/jbc.M112.430066 23184937 PMC3561522

[B157] Xie Y. Zhou X. Li J. Yao X. C. Liu W. L. Kang F. H. (2021). Identification of a new natural biflavonoids against breast cancer cells induced ferroptosis via the mitochondrial pathway. Bioorg. Chem. 109, 104744. 10.1016/j.bioorg.2021.104744 33639365

[B158] Xu J. Lamouille S. Derynck R. (2009). TGF-beta-induced epithelial to mesenchymal transition. Cell Res. 19, 156–172. 10.1038/cr.2009.5 19153598 PMC4720263

[B159] Yang F. Takagaki Y. Yoshitomi Y. Ikeda T. Li J. Kitada M. (2019). Inhibition of dipeptidyl peptidase-4 accelerates epithelial-mesenchymal transition and breast cancer metastasis via the CXCL12/CXCR4/mTOR Axis. Cancer Res. 79, 735–746. 10.1158/0008-5472.CAN-18-0620 30584072

[B160] Yang H. Yu N. Xu J. Ding X. Deng W. Wu G. (2018). SMURF1 facilitates estrogen receptor ɑ signaling in breast cancer cells. J. Exp. and Clin. cancer Res. CR 37, 24. 10.1186/s13046-018-0672-z 29433542 PMC5808446

[B161] Yang L. Bhattacharya A. Peterson D. Li Y. Liu X. Marangoni E. (2024b). Targeted dual degradation of HER2 and EGFR obliterates oncogenic signaling, overcomes therapy resistance, and inhibits metastatic lesions in HER2-positive breast cancer models. Drug Resist. Updat. Rev. Comment. Antimicrob. anticancer Chemother. 74, 101078. 10.1016/j.drup.2024.101078 PMC11070302 38503142

[B162] Yang Y. Luo M. Zhang K. Zhang J. Gao T. Connell D. O. (2020). Nedd4 ubiquitylates VDAC2/3 to suppress erastin-induced ferroptosis in melanoma. Nat. Commun. 11, 433. 10.1038/s41467-020-14324-x 31974380 PMC6978386

[B163] Yang Y. Wu M. Pan Y. Hua Y. He X. Li X. (2024a). WW domains form a folded type of nuclear localization signal to guide YAP1 nuclear import. J. Cell Biol. 223. 10.1083/jcb.202308013 PMC10942854 38488622

[B164] Yeung B. Ho K. C. Yang X. (2013). WWP1 E3 ligase targets LATS1 for ubiquitin-mediated degradation in breast cancer cells. PloS one 8, e61027. 10.1371/journal.pone.0061027 23573293 PMC3616014

[B165] Yim E. K. Peng G. Dai H. Hu R. Li K. Lu Y. (2009b). Rak functions as a tumor suppressor by regulating PTEN protein stability and function. Cancer Cell 15, 304–314. 10.1016/j.ccr.2009.02.012 19345329 PMC2673492

[B166] Yim E. K. Siwko S. Lin S. Y. (2009a). Exploring Rak tyrosine kinase function in breast cancer. Cell cycleGeorget. Tex. 8, 2360–2364. 10.4161/cc.8.15.9264 19597351

[B167] Yu J. M. Sun W. Wang Z. H. Liang X. Hua F. Li K. (2019). TRIB3 supports breast cancer stemness by suppressing FOXO1 degradation and enhancing SOX2 transcription. Nat. Commun. 10, 5720. 10.1038/s41467-019-13700-6 31844113 PMC6915745

[B168] Yu L. Liu X. Cui K. Di Y. Xin L. Sun X. (2015). SND1 acts downstream of TGFβ1 and upstream of Smurf1 to promote breast cancer metastasis. Cancer Res. 75, 1275–1286. 10.1158/0008-5472.CAN-14-2387 25596283

[B169] Zamore P. D. Haley B. (2005). Ribo-gnome: the big world of small RNAs. Sci. (New York, N.Y.) 309, 1519–1524. 10.1126/science.1111444 16141061

[B170] Zhang H. Zhang L. He Y. Jiang D. Sun J. Luo Q. (2024b). PI3K PROTAC overcomes the lapatinib resistance in PIK3CA-mutant HER2 positive breast cancer. Cancer Lett. 598, 217112. 10.1016/j.canlet.2024.217112 38986734

[B171] Zhang L. Qin Y. Wu G. Wang J. Cao J. Wang Y. (2020). PRRG4 promotes breast cancer metastasis through the recruitment of NEDD4 and downregulation of Robo1. Oncogene 39, 7196–7208. 10.1038/s41388-020-01494-7 33037408

[B172] Zhang L. Zhou F. García de Vinuesa A. de Kruijf E. M. Mesker W. E. Hui L. (2013). TRAF4 promotes TGF-β receptor signaling and drives breast cancer metastasis. Mol. Cell 51, 559–572. 10.1016/j.molcel.2013.07.014 23973329

[B173] Zhang S. Xiao X. Yi Y. Wang X. Zhu L. Shen Y. (2024a). Tumor initiation and early tumorigenesis: molecular mechanisms and interventional targets. Signal Transduct. Target. Ther. 9, 149. 10.1038/s41392-024-01848-7 38890350 PMC11189549

[B174] Zhang X. Jin T. G. Yang H. DeWolf W. C. Khosravi-Far R. Olumi A. F. (2004). Persistent c-FLIP(L) expression is necessary and sufficient to maintain resistance to tumor necrosis factor-related apoptosis-inducing ligand-mediated apoptosis in prostate cancer. Cancer Res. 64, 7086–7091. 10.1158/0008-5472.CAN-04-1498 15466204

[B175] Zhang X. Pickin K. A. Bose R. Jura N. Cole P. A. Kuriyan J. (2007). Inhibition of the EGF receptor by binding of MIG6 to an activating kinase domain interface. Nature 450, 741–744. 10.1038/nature05998 18046415 PMC3561764

[B176] Zhang X. Yalcin S. Lee D. F. Yeh T. Y. Lee S. M. Su J. (2011). FOXO1 is an essential regulator of pluripotency in human embryonic stem cells. Nat. Cell Biol. 13, 1092–1099. 10.1038/ncb2293 21804543 PMC4053529

[B177] Zhang Y. Chang C. Gehling D. J. Hemmati-Brivanlou A. Derynck R. (2001). Regulation of Smad degradation and activity by Smurf2, an E3 ubiquitin ligase. Proc. Natl. Acad. Sci. U. S. A. 98, 974–979. 10.1073/pnas.98.3.974 11158580 PMC14694

[B178] Zhang Y. E. (2009). Non-Smad pathways in TGF-beta signaling. Cell Res. 19, 128–139. 10.1038/cr.2008.328 19114990 PMC2635127

[B179] Zhang Z. Lu Y. X. Liu F. Sang L. Shi C. Xie S. (2023). lncRNA BREA2 promotes metastasis by disrupting the WWP2-mediated ubiquitination of Notch1. Proc. Natl. Acad. Sci. U. S. A. 120, e2206694120. 10.1073/pnas.2206694120 36795754 PMC9974429

[B180] Zhao B. Li L. Lu Q. Wang L. H. Liu C. Y. Lei Q. (2011). Angiomotin is a novel Hippo pathway component that inhibits YAP oncoprotein. Genes and Dev. 25, 51–63. 10.1101/gad.2000111 21205866 PMC3012936

[B181] Zhao B. Wei X. Li W. Udan R. S. Yang Q. Kim J. (2007). Inactivation of YAP oncoprotein by the Hippo pathway is involved in cell contact inhibition and tissue growth control. Genes and Dev. 21, 2747–2761. 10.1101/gad.1602907 17974916 PMC2045129

[B182] Zhao D. Zhi X. Zhou Z. Chen C. (2012). TAZ antagonizes the WWP1-mediated KLF5 degradation and promotes breast cell proliferation and tumorigenesis. Carcinogenesis 33, 59–67. 10.1093/carcin/bgr242 22045023

[B183] Zhao L. Zhao J. Zhong K. Tong A. Jia D. (2022). Targeted protein degradation: mechanisms, strategies and application. Signal Transduct. Target. Ther. 7, 113. 10.1038/s41392-022-00966-4 35379777 PMC8977435

[B184] Zheng H. Q. Zhou Z. Huang J. Chaudhury L. Dong J. T. Chen C. (2009). Krüppel-like factor 5 promotes breast cell proliferation partially through upregulating the transcription of fibroblast growth factor binding protein 1. Oncogene 28, 3702–3713. 10.1038/onc.2009.235 19668233

[B185] Zheng J. Shi Z. Yang P. Zhao Y. Tang W. Ye S. (2022). ERK-Smurf1-RhoA signaling is critical for TGFβ-drived EMT and tumor metastasis. Life Sci. alliance 5, e202101330. 10.26508/lsa.202101330 35654587 PMC9163791

[B186] Zhi X. Zhao D. Zhou Z. Liu R. Chen C. (2012). YAP promotes breast cell proliferation and survival partially through stabilizing the KLF5 transcription factor. Am. J. pathology 180, 2452–2461. 10.1016/j.ajpath.2012.02.025 22632819

[B187] Zhong C. Zhu R. Jiang T. Tian S. Zhao X. Wan X. (2024). Design and characterization of a novel eEF2K degrader with potent therapeutic efficacy against triple-negative breast cancer. Adv. Sci. Weinheim, Baden-Wurttemberg, Ger. 11, e2305035. 10.1002/advs.202305035 PMC10837347 38084501

[B188] Zhou F. Li F. Xie F. Zhang Z. Huang H. Zhang L. (2014). TRAF4 mediates activation of TGF-β signaling and is a biomarker for oncogenesis in breast cancer. Life Sci. 57, 1172–1176. 10.1007/s11427-014-4727-x 25249198

[B189] Zhou F. Xie F. Jin K. Zhang Z. Clerici M. Gao R. (2017). USP4 inhibits SMAD4 monoubiquitination and promotes activin and BMP signaling. EMBO J. 36, 1623–1639. 10.15252/embj.201695372 28468752 PMC5452037

[B190] Zhou L. Li H. Sun T. Wen X. Niu C. Li M. (2022). HULC targets the IGF1R-PI3K-AKT axis in trans to promote breast cancer metastasis and cisplatin resistance. Cancer Lett. 548, 215861. 10.1016/j.canlet.2022.215861 35981570

[B191] Zhou Z. Liu R. Chen C. (2012). The WWP1 ubiquitin E3 ligase increases TRAIL resistance in breast cancer. Int. J. cancer 130, 1504–1510. 10.1002/ijc.26122 21480222

[B192] Zhu H. Kavsak P. Abdollah S. Wrana J. L. Thomsen G. H. (1999). A SMAD ubiquitin ligase targets the BMP pathway and affects embryonic pattern formation. Nature 400, 687–693. 10.1038/23293 10458166

[B193] Zhu K. Shan Z. Chen X. Cai Y. Cui L. Yao W. (2017). Allosteric auto-inhibition and activation of the Nedd4 family E3 ligase Itch. EMBO Rep. 18, 1618–1630. 10.15252/embr.201744454 28747490 PMC5579370

[B194] Zhu S. Wu Y. Song B. Yi M. Yan Y. Mei Q. (2023). Recent advances in targeted strategies for triple-negative breast cancer. J. Hematol. and Oncol. 16, 100. 10.1186/s13045-023-01497-3 37641116 PMC10464091

